# Impact and cost-effectiveness of water, sanitation, hygiene, and health education interventions in the control and elimination of soil-transmitted helminthiasis: A scoping review

**DOI:** 10.1371/journal.pntd.0014664

**Published:** 2026-08-25

**Authors:** Collins Okoyo, Michael Odhiambo, Geofrey Chepchieng, John Kioko, Britney Nyanchama, Michelle Anjela, Charles Mwandawiro, Mark Minnery

**Affiliations:** 1 Eastern and Southern Africa Centre of International Parasite Control, Kenya Medical Research Institute, Nairobi, Kenya; 2 Department of Epidemiology, Statistics and Informatics, Kenya Medical Research Institute, Nairobi, Kenya; 3 Deworm the World Initiative, Evidence Action, Nairobi, Kenya; Consejo Nacional de Investigaciones Cientificas y Tecnicas, Fundación Mundo Sano, ARGENTINA

## Abstract

**Background:**

Soil-transmitted helminthiasis (STH) remains a major public health problem in many low- and middle-income countries (LMICs) despite widespread implementation of mass drug administration (MDA). Rapid reinfection after treatment highlights the limitations of MDA approaches and underscores the need for complementary interventions. Water, sanitation, hygiene, and health education (WASH) interventions are widely promoted as essential components of integrated STH control strategies. However, evidence on WASH impact and cost-effectiveness remains fragmented and is poorly synthesized. This scoping review aimed to systematically map and summarize the evidence on the impact and cost-effectiveness of WASH interventions for STH control and elimination.

**Methods:**

Following Arksey and O’Malley’s framework and the Preferred Reporting Items for Systematic reviews and Meta-Analysis extension for Scoping Reviews (PRISMA-ScR) guidelines, we conducted a scoping review of peer-reviewed and grey literature with no date restrictions. We searched PubMed and Google Scholar for papers that met our inclusion criteria. Studies assessing the impact and/or cost-effectiveness of WASH interventions, alone or integrated with MDA, on STH outcomes were included.

**Principal findings:**

Sixteen studies, predominantly from LMICs, met the inclusion criteria, with Kenya contributing the largest number. Findings showed that combining WASH interventions with MDA led to greater reductions in reinfection with *Ascaris lumbricoides* than MDA alone, particularly in school- and community-based programs. However, behavioural improvements alone were insufficient without supporting infrastructure, and their effects on other helminths were inconsistent. Notably, only one study conducted a formal cost-effectiveness analysis of WASH interventions, revealing a critical gap in economic evidence.

**Conclusion:**

Integrating WASH interventions with MDA can enhance STH control outcomes, particularly for *A. lumbricoides,* but the lack of robust economic evaluations limits informed policy and investment decisions. Future research should prioritize cost-effectiveness analyses and long-term assessments of integrated WASH strategies to support sustainable STH elimination efforts.

## Author summary

We carried out a scoping review to examine how water, sanitation, hygiene, and health education (WASH) interventions influence soil-transmitted helminthiasis (STH) and whether they are cost-effective. Using a systematic approach, we identified 16 studies, most from low- and middle-income countries where WASH services are often limited. The majority of studies focused on roundworm (*Ascaris lumbricoides*), with fewer addressing whipworm (*Trichuris trichiura*) and hookworm. We found that combining WASH interventions with mass drug administration (MDA) improved control of *Ascaris lumbricoides* compared to MDA alone. Hygiene education by itself was rarely enough without improved sanitation or water infrastructure, and the effects on other STH parasites were mixed. Very few studies assessed costs or cost-effectiveness, leaving important gaps in evidence for guiding investments. Our findings point to the need for integrating WASH strategies into routine MDA programs and for stronger collaboration among governments, non-governmental organizations, and communities. Expanding economic research will be essential to support decisions that can reduce infections and improve health outcomes. By highlighting these gaps, we provide evidence that can inform sustainable approaches to controlling and eventually eliminating STH in vulnerable populations.

## 1. Introduction

Soil-transmitted helminthiasis (STH), namely *Ascaris lumbricoides*, *Trichuris trichiura*, and hookworms (*Necator americanus* and *Ancylostoma duodenale*), remains a significant public health concern, particularly in low- and middle-income countries (LMICs). These parasites are transmitted by ingestion of eggs from contaminated soil (*A. lumbricoides* and *T. trichiura*) or by larval penetration of the skin (hookworms). STH transmission is exacerbated by poor water, sanitation, and hygiene conditions, and limited health education (WASH) [1,2]. Routine mass drug administration (MDA) programs, particularly school-based platforms, have led to substantial reductions in STH infections. Nonetheless, reinfection remains a concern in many endemic countries. Studies have reported reinfection rates of up to 10.4% within 1 year after 5 rounds of MDA [3].

Vulnerable groups for STH infections include preschool and school-aged children, as well as women of reproductive age in rural areas, who experience the highest prevalence and intensity of infections [2,4]. Control efforts primarily rely on preventive chemotherapy with a single dose of albendazole or mebendazole, administered annually or biannually based on community prevalence thresholds [5,6]. Despite these gains, the rapid reinfection underscores the urgent need to integrate WASH with MDA to achieve sustainable interruption of transmission.

Control and eventual elimination of STH is of critical importance due to their substantial impact on public health, education, and economic development. Intestinal helminths contribute to malnutrition, iron-deficiency anaemia, growth retardation, and impaired cognitive development, particularly among school-aged children [1,7]. These health effects result in poor school attendance and academic performance, perpetuating the cycle of poverty in endemic areas. Although MDA programs have significantly reduced prevalence in many endemic countries, reinfection remains a major concern, highlighting the need for more sustainable and comprehensive control strategies.

Integrating WASH interventions offers a more holistic, long-term solution to helminth control. While MDA can reduce infection levels quickly, its effects are often temporary unless accompanied by improvements in living conditions and personal hygiene [8]. WASH infrastructure, including access to clean water and improved sanitation, is vital for interrupting transmission pathways, while health education encourages lasting behavioural change. By reducing reinfection rates, these combined efforts not only protect vulnerable populations but also support progress towards the World Health Organization (WHO) 2030 neglected tropical disease (NTD) elimination targets [6].

Health education plays a complementary role by promoting behaviour change and increasing community awareness of STH prevention. Educating children and caregivers about hand hygiene, safe defecation, food safety, and the danger of open defecation fosters healthier practices and reinforces the benefits of WASH infrastructure [8,9]. School-based health education programs, in particular, have shown success in improving hygiene behaviours and reducing reinfection rates among children, the primary risk group for STH [2].

Despite decades of efforts targeting STH control, including school-based MDA programs and selective WASH interventions, reinfection rates remain high in many endemic countries, and evidence of long-term sustainability is limited. For example, in Kenya, five rounds of MDA resulted in significant reductions in prevalence, but persistent regional heterogeneity suggested retransmission due to environmental exposure and varying WASH conditions [10,11]. While cluster-randomized trials show that integrating school-based WASH interventions with deworming can reduce reinfection [8], these have not been accompanied by economic analyses assessing cost-effectiveness.

While significant economic evaluations have largely focused on drug delivery strategies, such as comparisons between community-wide and school-based MDA, these studies have reported mixed findings regarding effectiveness and equity [12]. There are far fewer studies that specifically assess the combined viability of WASH interventions across diverse transmission contexts.

While this scoping review does not test a formal statistical hypothesis due to its exploratory nature, it is grounded in a clear framework that outlines the expected links between the interventions and epidemiological outcomes. Standard preventive chemotherapy through MDA targets the individual parasite burden, leading to quick, temporary drops in prevalence and intensity. On the other hand, integrated WASH interventions aim to disrupt the environmental pathways through which STH eggs and larvae spread. Specifically, infrastructure elements such as safe water supply and improved sanitation facilities should reduce environmental faecal contamination. Meanwhile, behavioural aspects, such as promoting hand hygiene and providing health education, should reduce human contact with contaminated soil and water. Therefore, this integrated strategy is expected to show a combined effect, with the addition of WASH interventions to MDA resulting in lower reinfection rates and more sustained declines in STH prevalence than MDA alone can achieve.

With growing investment from both government and international agencies in WASH integration, it is imperative to map the existing literature on the impact and cost-effectiveness of WASH interventions on STH to inform resource allocation, identify evidence gaps, and guide the development of scalable models. Therefore, this study conducted a focused scoping review to provide critical insights for policymakers and donors seeking to optimize integrated WASH strategies in line with the WHO 2030 NTD targets.

## 2. Materials and methods

A review protocol for this study was published in the Open Science Framework on November 2, 2025, and can be accessed at (https://osf.io/kpmx3/files/uqb4e). The writing of the manuscript of this scoping review followed the Preferred Reporting Items for Systematic reviews and Meta-Analysis extension for Scoping Reviews (PRISMA-ScR) guidelines. The PRIMA-ScR checklist is available in the S1 Checklist. An initial search strategy and selection criteria were developed to guide this scoping review. This review was conducted to produce focused research in a timely manner. It followed the outline of Arksey and O’malley’s framework [13] by carefully focusing on the question, using broader search strategies, and extracting only key variables. The title, abstract, and full-text screening were conducted independently by two reviewers using predefined eligibility criteria guided by the Population-Concept-Context (PCC) framework. Discrepancies were resolved through discussion and consensus, with involvement of a third reviewer where necessary. Formal inter-rater statistics were not calculated, consistent with the exploratory nature of scoping reviews.

### 2.1 Research question

The primary research question was: *What is the impact and cost-effectiveness of water, sanitation, hygiene, and health education in the control and elimination of soil-transmitted helminthiasis as measured by epidemiological outcomes (infection prevalence, infection intensity, reinfection, etc.), economic costs, and cost-effectiveness outcomes (incremental cost-effectiveness ratio, quality-adjusted life years, etc.)?*

### 2.2 Inclusion and exclusion criteria

Studies were included based on the following criteria: (1) The *target populations*: preschool aged children, school-aged children, and women of reproductive age, (2) The *geographical scope*: global, (3) The *time frame*: unrestricted, (4) The *content focus*: studies that focused on WASH interventions and STH, and (5) *The source types*: peer-reviewed journal articles and relevant grey literature reports meeting the primary study eligibility criteria. The exclusion criteria excluded studies that did not implement WASH interventions, did not address STH, or were systematic reviews or meta-analyses without primary data. This inclusion/exclusion framework aligns with the PCC mnemonic recommended by the Joanna Briggs Institute (JBI) for scoping review studies [14].

### 2.3 Databases searched

An internet-based literature search was performed. Databases searched for published materials were PubMed and Google Scholar. Grey literature was identified through manual searches of key organizational websites, including WHO, Institutional Repository for Information Sharing (IRIS), the Children’s Investment Fund Foundation (CIFF), and Evidence Action’s technical repositories. Institutional repositories, including the Kenya Medical Research Institute (KEMRI) digital library, were also searched. Conference proceedings reviewed included the American Society of Tropical Medicine and Hygiene (ASTMH) annual meetings, National NTD Programme Managers annual meetings, the KEMRI Annual Scientific and Health Conference (KASH), and the first International Conference on NTDs in Kenya. Reference lists of all included studies were manually screened. These searches were conducted using a combination of the predefined keywords to ensure consistency. All database, grey literature, and repository searches were conducted collaboratively by the review team between June 2025 and September 2025.

### 2.4 Search terms or keywords

The keywords used for the literature search were “water **OR** sanitation **OR** hygiene **OR** health education” **AND** “soil-transmitted helminths **OR** soil-transmitted helminthiasis **OR** intestinal helminthiasis **OR** *Ascaris lumbricoides* **OR** *Trichuris trichiura* **OR** hookworm **OR** *Necator americanus* **OR** *Ancylostoma duodenale*”, **OR** “public health measures **OR** intestinal helminth control interventions **OR** soil-transmitted helminth control interventions **OR** water, sanitation and hygiene interventions **OR** health education interventions **OR** auxiliary interventions **OR** complementary interventions”, **OR** “cost **OR** effectiveness **OR** cost-effectiveness **OR** cost-effective interventions”, **OR** “mass drug administration”.

### 2.5 Searching strategy

Literature searches followed the standard three-step approach described in the JBI guidelines [14]. The aim was to identify all relevant studies reporting on the impact and/or cost-effectiveness of WASH interventions in relation to STHs. In the first step, decisions were made regarding which databases to use. Based on preliminary scoping exercises and similar reviews in the field, PubMed was identified as the primary academic database due to its wide coverage of peer-reviewed public health and infectious disease studies. Additionally, Google Scholar was selected to increase coverage of multidisciplinary and cross-sectoral sources relevant to WASH interventions and economic evaluation.

Although the search was limited to PubMed and Google Scholar, these databases were selected due to their broad coverage of biomedical and interdisciplinary literature. Google Scholar was particularly useful for capturing grey literature and non-indexed sources relevant to WASH and economic evaluations. The second step involved conducting structured database searches using the defined search terms**.** In the third step, backward citation tracking was conducted by reviewing the reference lists of all relevant primary studies and reviews retrieved in step two. This process helped identify additional literature not captured through database indexing but relevant to the topic.

### 2.6 Study selection and screening

**Fig 1:**
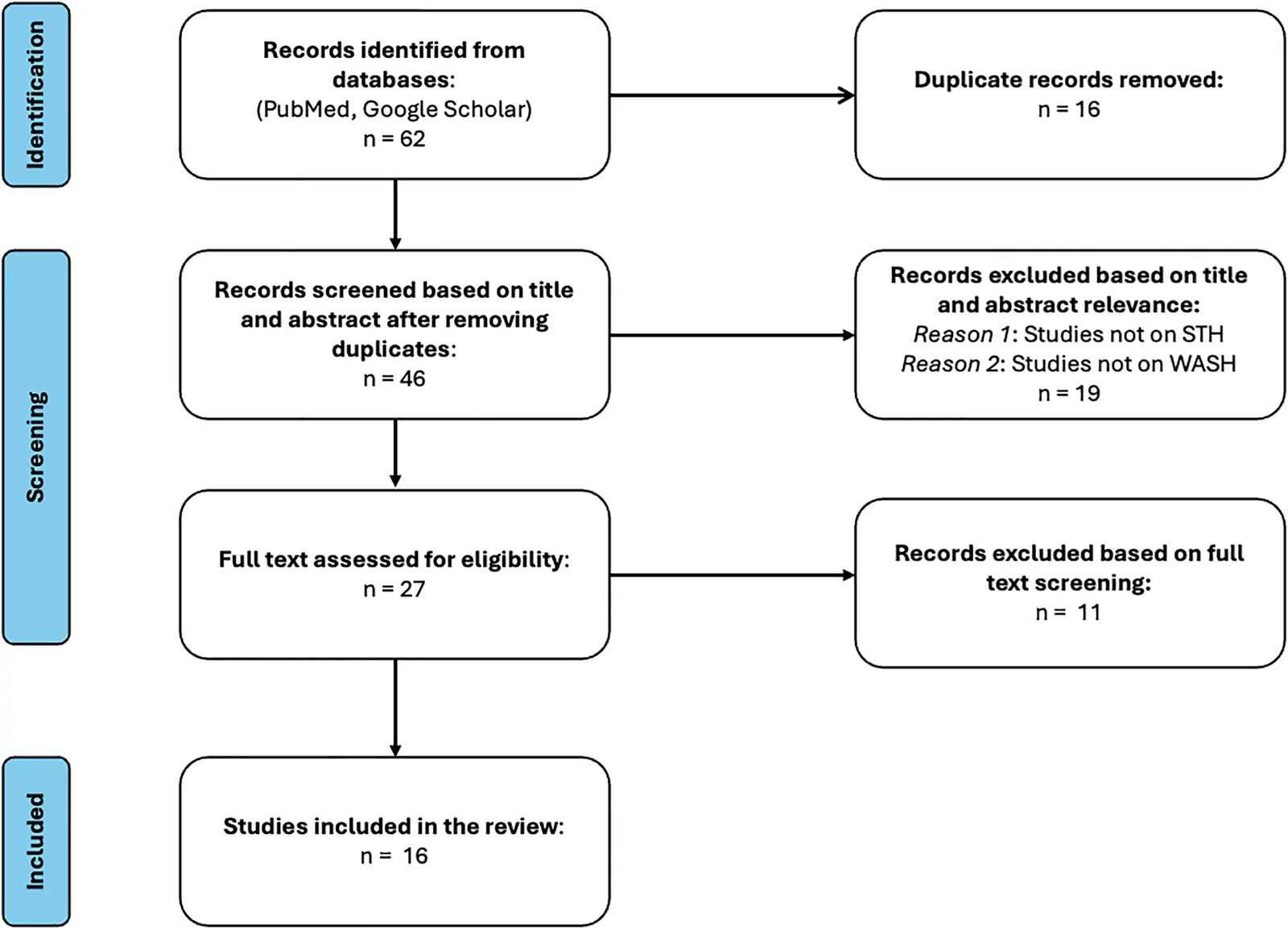
The selection process for studies included in the scoping review.

The selection of studies for inclusion in this scoping review followed a two-stage screening process: Title and abstract screening, followed by full-text screening. In the first stage, all records identified through the chosen database searches (i.e., PubMed and Google Scholar) were downloaded into a folder in Mendeley Reference Manager. We then screened the titles and abstracts of all retrieved citations against the eligibility criteria defined in the PCC framework. Studies were excluded at this stage if they were clearly irrelevant or did not focus on WASH interventions in relation to STH. In the second stage, we retrieved the full texts of all potentially eligible studies and reviewed them in detail against the pre-specified eligibility criteria (Fig 1).

### 2.7 Data extraction and analysis

Data extraction was performed by MO, GC, JK, BN, and MA, and the data were reviewed by CO, CM, and MM. The information extracted from the studies included: first author’s name and year of publication, country of study, study title, study objectives, study population or setting, study design, interventions implemented, and key findings related to the interventions (S1 Table).

## 3. Results

### 3.1 Geographical distribution

A total of 16 studies were included in this scoping review. These studies represented a range of designs, including seven cluster-randomized controlled trials (44%), four observational cross-sectional studies (25%), two mathematical modelling studies (12%), and three mixed or quasi-experimental studies (19%). Across the included studies, the primary STH parasites assessed were *A. lumbricoides, T. trichiura,* and hookworms. *A. lumbricoides* was the most frequently reported, while fewer studies reported outcomes for *T. trichiura* and hookworms. Geographically, the studies were conducted across various low- and middle-income countries, with Kenya contributing the most (6 studies, 38%), followed by Ethiopia (4 studies, 25%), Timor-Leste (3 studies, 19%), and one study each (6%) from Laos, Bangladesh, and Thailand. Although extensive searches of grey literature repositories and institutional databases were conducted, no grey literature records met all inclusion criteria for final extraction and analysis. As a result, the final synthesis included 16 peer-reviewed studies.

### 3.2 Variation in interventions

Across the included studies, there was substantial diversity in both MDA and WASH intervention design. MDA regimens ranged from single-dose administration to routine annual delivery embedded within national programs [15], as well as high-frequency strategies modelled at 3- or 6-month intervals [16]. Delivery platforms also varied, with some interventions targeting school-aged children exclusively [8,17], while others adopted community-wide approaches that included adults [18,19].

Similarly, WASH interventions ranged from education-only approaches focused on behaviour change [20] to comprehensive packages that incorporated water treatment, sanitation infrastructure, handwashing facilities, and hygiene promotion [18,21]. Several studies also evaluated individual WASH components separately (e.g., water treatment, sanitation, or handwashing alone), enabling comparison of their relative effects.

### 3.3 Comparative effectiveness of WASH components

Clear differences emerged in the effectiveness of WASH intervention types. Studies combining infrastructure improvements with behavioural interventions generally reported more consistent reductions in infection, particularly for *A. lumbricoides.* For example, cluster-randomized trials in Kenya and Bangladesh [21,22] showed that integrated WASH packages (water, sanitation, and handwashing) achieved greater reductions in *A. lumbricoides* prevalence than single-component interventions or control groups.

In contrast, education-only interventions, although effective at improving hygiene practices, had limited impact on infection outcomes. In Ethiopia, Gizaw and colleagues [20] reported substantial improvements in handwashing and sanitation behaviours, but no statistically significant reduction in infection prevalence. Similarly, studies relying on hygiene promotion without corresponding infrastructure improvements showed weaker or inconsistent epidemiological effects.

While these observed differences highlight general patterns across intervention typologies, formal head-to-head evaluations of comparative efficacy or intervention quality across these heterogeneous studies fall outside the exploratory mapping mandate of this scoping review.

### 3.4 School vs community-level interventions

The level at which interventions were implemented also influenced outcomes. School-based WASH interventions, such as those evaluated in Laos [17] and Kenya [8], improved hygiene practices and, in some cases, reduced reinfection with *A. lumbricoides*. However, their overall impact on infection prevalence was inconsistent, particularly when household and environmental exposure remained unchanged.

In contrast, community-wide interventions targeting both children and adults showed potential for broader impact on transmission dynamics. For example, the WASH for WORMS trial in Timor-Leste [19] achieved significant reductions in open defecation but did not observe corresponding reductions in infection during the study period. This underscores that although interventions often focus on high-risk demographic subgroups, interrupting transmission requires shifting the dynamics of the entire community, as unmonitored populations can maintain environmental reservoirs of infection. Similarly, pilot findings from Timor-Leste [18] suggested that community-level interventions may yield greater reductions in infection than school-based approaches alone, although these results were not statistically conclusive. These findings highlight the importance of addressing transmission at the community level rather than focusing solely on schools.

### 3.5 Parasite-specific effects

Across studies, intervention effects varied by STH parasites. Reductions in *A. lumbricoides* were the most consistently reported outcome, particularly in studies combining WASH and MDA [8,21]. In contrast, evidence for *T. trichiura* and hookworm was more limited and inconsistent. Several studies reported no significant effects for these parasites, often because of low baseline prevalence or differences in transmission pathways [8,22]. These findings suggest that WASH interventions may vary in effectiveness depending on parasite biology and environmental persistence.

### 3.6 Gaps in the evidence

Evidence on cost-effectiveness was extremely limited, with only one study [23] conducting a formal economic evaluation. The study found that chemotherapy-only strategies were more cost-effective than those incorporating health education, despite improvements in knowledge and behaviour. However, this evidence is outdated and does not reflect contemporary integrated WASH approaches that include infrastructure components. The absence of cost-effectiveness data from more recent large-scale trials and integrated interventions represents a critical gap, particularly given the substantial investment required for WASH implementation.

## 4. Discussion

This scoping review synthesizes evidence on the impact and cost-effectiveness of water, sanitation, hygiene, and health education interventions integrated with MDA to control and eliminate STH. Overall, the findings demonstrate that integrated WASH approaches can improve STH control outcomes compared with MDA alone, particularly for *A. lumbricoides.* The review also reveals substantial gaps in economic evaluation, long-term effectiveness, and geographical coverage.

Recent primary research supports integrating WASH education into drug-based control strategies. A quasi-experimental study of Karen hill tribe communities in northern Thailand found that combining MDA with targeted WASH significantly reduced reinfection with intestinal parasites compared with MDA alone and was associated with significant improvements in participants’ knowledge, attitudes, and practices related to infection prevention [15]. Although the study did not evaluate comprehensive infrastructure improvements, the findings suggest that behaviour-focused WASH can enhance the short-term effectiveness of STH control efforts when paired with chemotherapy, particularly in resource-limited settings where poor hygiene practices and environmental exposure drive reinfection.

Observational evidence from the Geshiyaro project in Wolayita, Ethiopia, further underscores the importance of WASH access in controlling STH transmission. Using population-based cross-sectional data [24], it was demonstrated that both individual- and community-level access to improved water, sanitation, and hygiene were associated with a lower prevalence of *A. lumbricoides, T. trichiura,* and hookworm infections. Notably, community-level sanitation coverage showed stronger and more consistent associations with reduced infection than household-level access alone, suggesting that shared environmental exposure plays a critical role in sustaining transmission.

While randomized trials such as WASH for WORMS have significantly advanced understanding of the epidemiological impact of integrated WASH and deworming programs, they also highlight a persistent gap in economic evidence on cost-effectiveness. The WASH for WORMS cluster-randomized controlled trial in rural Timor-Leste found that a community-based WASH intervention integrated with periodic deworming did not yield additional reductions in STH infections beyond those achieved by deworming alone over a two-year period, despite substantial improvements in sanitation practices in the intervention arm [19].

In a community-based study in rural Bangladesh [23], a single round of albendazole was more cost-effective than regimens that included regular health education alongside chemotherapy for reducing the combined prevalence and intensity of *A. lumbricoides, T. trichiura,* and hookworm infections, suggesting that adding educational components did not improve cost-effectiveness in that setting. Although this study predates many contemporary WASH and integrated control strategies and did not evaluate comprehensive infrastructure improvements, it illustrates that health education alone may not offer cost advantages when combined with deworming and highlights the importance of rigorous economic analyses when considering additional WASH components.

Findings vary across studies, likely reflecting a combination of epidemiological and implementation-related factors. Differences in baseline transmission intensity, sanitation coverage, population density, and adherence to interventions may substantially affect the effectiveness of integrated WASH and MDA strategies. For example, in high-transmission settings with widespread environmental contamination, short-term WASH interventions may not be sufficient to produce measurable reductions in infection, even when improvements in hygiene behavior are observed [19,20]. In contrast, studies in settings with higher WASH intervention coverage have shown more pronounced reductions in infection [8,21]. These findings suggest that the success of WASH interventions is highly context-dependent and underscore the need for sustained, high-coverage implementation.

From a policy perspective, these findings reinforce the need to move beyond reliance on MDA alone and toward integrated, long-term strategies that address environmental transmission. While MDA provides rapid reductions in the infection burden, sustainable control will likely require investment in sanitation infrastructure, access to safe water, and sustained behavior-change interventions. However, the limited availability of cost-effectiveness evidence makes it difficult for policymakers to prioritize and scale these interventions, particularly in resource-constrained settings.

A key limitation of this scoping review is the lack of evidence on the costs and cost-effectiveness of integrating WASH interventions with deworming programs for STH control. Although numerous studies assess epidemiological outcomes, very few included economic analyses, and those that do are often outdated or limited in scope. This limits the ability to assess the value for money of integrated approaches and to compare them with standard MDA strategies, particularly in resource-constrained settings.

Another limitation concerns the screening process. Although study selection was conducted independently by two reviewers using predefined eligibility criteria, formal pilot testing and quantitative inter-rater reliability assessment were not conducted. Although discrepancies were resolved through discussion and consensus, the absence of pilot testing may have introduced minor inconsistencies in the interpretation of the eligibility criteria. However, the use of clearly defined PCC-based inclusion and exclusion criteria minimized this risk. In addition, the review focused primarily on key at-risk groups (children and women of reproductive age). While this aligns with standard WHO target frameworks, limiting the primary synthesis to these two cohorts may constrain the generalizability of our findings on community-wide transmission dynamics, where untreated or unmonitored segments of the broader population continue to contribute to environmental parasite contamination.

Finally, the review was limited by its reliance on two primary databases, PubMed and Google Scholar, for the peer-reviewed literature search. Although these databases provide extensive coverage of public health and economic research, some relevant studies indexed exclusively in other regional or specialized databases may have been missed. To mitigate this limitation, a comprehensive grey literature search was conducted across multiple institutional repositories, complemented by thorough backward citation tracking of all included articles.

## 5. Conclusion

Integrated WASH and MDA interventions show potential to enhance STH control and elimination, and reduce reinfection, particularly for *A. lumbricoides.* However, the strength of the evidence varies across settings and intervention types, and remains limited for certain parasites and outcomes. Further high-quality studies, including robust economic evaluations, are needed to better understand the effectiveness and cost-effectiveness of these approaches and to inform context-specific policy and implementation.

## Supporting information

S1 Checklist The Preferred Reporting Items for Systematic reviews and Meta-Analysis extension for Scoping Reviews (PRISMA-ScR) checklist. https://doi.org/10.7326/M18-0850. Link: https://www.prisma-statement.org/scoping.
(DOCX)

S1 Table Characteristics of the studies included and analyzed in this scoping review. (DOCX)
