# Supplementary material for: Impact and cost-effectiveness of water, sanitation, hygiene, and health education interventions in the control and elimination of soil-transmitted helminthiasis: A scoping review
Source: PLoS Negl Trop Dis. 2026 Aug 25;20(8):e0014664. doi: 10.1371/journal.pntd.0014664 (PMC13529219; doi:10.1371/journal.pntd.0014664)
Supplement: Supplementary file 2 [file pntd.0014664.s002.docx]

**Impact and Cost-Effectiveness of Water, Sanitation, Hygiene, and Health Education Interventions in the Control and Elimination of Soil-Transmitted Helminthiasis: A Scoping Review**

Collins Okoyo^1,2*^, Michael Odhiambo^1^, Geofrey Chepchieng^1^, John Kioko^1^, Britney Nyanchama^1^, Michelle Anjela^1^, Charles Mwandawiro^1^, and Mark Minnery^3^

**Affiliations:**

^1^Eastern and Southern Africa Centre of International Parasite Control, Kenya Medical Research Institute, Nairobi, Kenya

^2^Department of Epidemiology, Statistics and Informatics, Kenya Medical Research Institute, Nairobi, Kenya

^3^Deworm the World Initiative, Evidence Action, Nairobi, Kenya

* [cokoyo@kemri.go.ke](mailto:cokoyo@kemri.go.ke)

**S1 Table:** Characteristics of the studies included and analyzed in this scoping review

| **No.** | **First author name & publication date** | **Study country** | **Title of the study** | **Objectives of the study** | **Study population / setting** | **Study design** | **Intervention(s)** | **Key findings** |
| --- | --- | --- | --- | --- | --- | --- | --- | --- |
| 1. | Nachaiwieng, et al., 2024 | Thailand | The impact of an integrated  intervention program combining  drug therapy with water, sanitation,  and hygiene (WASH) education on  reinfection with intestinal parasitic  infections among the Karen hill  tribe in northern Thailand | To evaluate whether integrating  WASH education with mass drug  administration (MDA) reduces  reinfection rates of intestinal  parasitic infections (IPIs) more  effectively than drug treatment  alone in a high-risk, underserved  population. | 691 participants from two Karen hill  tribe villages in northern Thailand. | Quasi-experimental, community-based  study with baseline and follow-up  assessments at 3 and 6 months. | Mass drug administration (MDA): Single dose of albendazole.  WASH education (intervention group only): Delivered through participatory methods  (lectures, leaflets, home visits).  Promoted hygiene practices and proper sanitation. | Baseline data revealed a 36.0% (123/342) prevalence of IPIs in the intervention group and 36.8% (96/261) in the control group. Post-intervention at 3-month and 6-month follow-ups revealed that the prevalence of IPIs in the intervention group was significantly decreased to 23.6% (p=0.002) and 23.1% (p=0.002), respectively. In contrast, no significant changes in prevalence of IPIs were observed in the control group. The intervention group showed significant improvements in knowledge, attitude and practices (KAP) scores, which were significantly higher than those in the control group. MDA alone is not effective for controlling IPIs due to rapid reinfection related to behavioral factors and socioeconomic conditions. The researchers demonstrated that integration of WASH education increased KAP scores and consequently significantly reduced IPI reinfection among the Karen hill tribe in northern Thailand. |
| 2. | Phillips, et al., 2022 | Ethiopia | Association between water, sanitation, and hygiene access and the prevalence of soil-transmitted helminth and schistosome infections in Wolayita,  Ethiopia | To assess the associations between  individual- and community-level access to water, sanitation, and hygiene (WASH) and  the prevalence of soil-transmitted helminths  (STHs) and schistosomiasis in southern  Ethiopia. | Wolayita Zone, Southern Ethiopia | Cross-sectional observational study. | The study itself did not implement new interventions. Instead, it analyzed data from an existing program - the Geshiyaro project - which included mass drug administration (MDA) and ongoing WASH interventions (like improved water access and  sanitation facilities). The study assessed how existing levels of WASH access (not newly  introduced treatments) were associated with the prevalence of helminth and schistosome infections in the population. | Shared latrines, lack of handwashing facilities, and  water sources >30 minutes away were associated with higher odds of helminth infections.  Community-level access to sanitation and water was more strongly associated with reduced  infection than individual household access.  Supports the role of WASH improvements, particularly at scale, in sustainable parasite control. |
| 3. | Okoyo, et al., 2021 | Kenya | Modeling the Interruption of the Transmission of Soil  -Transmitted Helminths Infections in Kenya: Modeling  Deworming, Water, and Sanitation Impacts | To develop an age‑structured mathematical model (based on ordinary differential equations) assessing how mass drug  administration (MDA) alone and in combination with WASH interventions influence worm burden and the time needed to interrupt soil-transmitted  helminth (STH) transmission in Kenya. | The study population included: Pre-school aged children (2–4 years), School-aged children (5–14 years) and Adults (≥15 years) | A deterministic, age-structured ODE model was developed.  Comparisons made include:  MDA-only strategies at different frequencies, MDA combined with high-coverage WASH interventions at different frequencies. | The interventions modeled included: Mass Drug Administration (MDA):  Scenarios of annual vs. 3-montly or 6-monthly dosing in different age groups.  WASH interventions: Improvements in water, sanitation, and hygiene with varying assumed coverage levels. | MDA alone significantly  reduced worm burden but did not show achievement of interruption within short time period unless delivered at very high frequency (e.g., 3-monthly  dosing).  Combining MDA with WASH, especially at ≥95% population coverage, can dramatically reduce time to interruption and substantially lower worm burden  across age groups.  Outcomes were found to be sensitive to four key parameters:  drug efficacy, frequency of MDA, MDA coverage, and WASH coverage. |
| 4. | Okoyo, et al., 2021 | Kenya | Statistical regression model of water, sanitation, and  hygiene; treatment coverage; and environmental  influences on school-level soil-transmitted helminths  and schistosome prevalence in Kenya: Secondary  analysis of the national deworming program data | To identify the key determinants of school-  level STH and schistosomiasis prevalence  and relative reduction in Kenyan schools  over five years (2012–2017), by modeling  effects of treatment rounds, school and  household WASH conditions, and  environmental factors using existing  national deworming program data | 199 schools across Kenya involved in the National School-Based Deworming  Program (NSBDP) | Secondary analysis of national program  data | No intervention. The study was a secondary data analysis using observational data from Kenya’s  National School-Based Deworming Program  (NSBDP) collected between 2012 and 2017. | *Ascaris lumbricoides:*  Larger relative reduction associated with baseline prevalence < 1%, seven treatment rounds of treatment, household handwashing coverage of 50–75%, mean land surface temperature (20-25 °C), and community density approximately 5-10 persons per 100 m²  *Hookworm:*  Greater reduction seen in settings with baseline prevalence < 1% and presence of a school feeding program  *Trichuris trichiura:*  Reduction primarily associated with baseline prevalence < 1%. |
| 5. | Gitore, et al., 2020 | Ethiopia | Prevalence of soil-transmitted helminths and its association with water, sanitation, hygiene among schoolchildren and  barriers for school level prevention in technology villages of Hawassa University: Mixed design | To determine the prevalence of soil-transmitted helminth (STH) infections among schoolchildren in the “technology villages”  of Southern Ethiopia, investigate associations with water, sanitation, and hygiene (WASH)  factors, and identify barriers at the school level to preventing STH transmission. | The study population was 1,080 schoolchildren in schools within the technology villages associated with Hawassa University, Southern Ethiopia. The study was carried out between September 5 – October 15, 2019. | Institution-based cross-sectional analytical study with a mixed  method design.  The design also included a survey questionnaire and analysis of  stool specimen.  In‑depth interviews with key informants including school staff and community stakeholders were also performed.  Sampling included a two-stage cluster sampling for quantitative participants and purposive  sampling for qualitative informants. | Collected stool specimens (~2g per child), analyzed via direct wet mount and Kato‑Katz techniques.  Administered a structured questionnaire on demographics,  WASH behaviors, and knowledge/practices.  Observational checklist for  school WASH infrastructure.  Conducted in-depth interviews  to identify institutional and  contextual barriers to school-level STH prevention. | *Prevalence of STHs:*  The overall prevalence of any STHs was 23.1% (95% CI: 21.4, 27.6). The most commonly  identified parasites were: *Ascaris lumbricoides,*  *Trichuris trichiura* and hookworms.  *Significant Risk Factors*:  Family Size: Children from households with more than five members were nearly twice as  likely to be infected with STHs.  Distance to Latrine: When latrines were located more than 100 meters away from the home or school, the likelihood of infection was significantly higher. Distance discourages frequent use of latrines and may lead to open defecation, a major transmission route for STHs.  *Hygiene and Sanitation Conditions:*  Observational data revealed that many schools lacked basic water and sanitation facilities. Handwashing stations, if present, often lacked soap or running water. Toilets were often poorly maintained, with shared or non-functional latrines. These conditions foster an environment where children can easily pick up infections  from contaminated surfaces or soil, especially during play or eating without washing hands.  *Qualitative Findings:* Institutional Barriers: Interviews with teachers and administrators  uncovered the following challenges:  Lack of WASH infrastructure and budget to maintain existing facilities. Insufficient health education related to hygiene. Overcrowded classrooms that  hinder hygiene promotion. Weak collaboration between schools and health offices in implementing deworming or hygiene education programs. |
| 6. | SV Nery, et al., 2019 | Timor-Leste | WASH for worms: A cluster-randomized controlled trial of the impact of a community integrated water, sanitation, and hygiene and deworming intervention on soil-transmitted helminth infections | To evaluate whether combining a community-wide WASH intervention with mass drug administration (MDA) is more effective in reducing soil-transmitted helminth (STH) infections than MDA alone. | Location: 18 rural communities in  Manufahi District, Timor-Leste.  Participants: Entire community populations, including children and adults. | Cluster-randomized controlled trial | The intervention implemented in this study was a comprehensive, community-integrated Water, Sanitation, and Hygiene (WASH) program designed to reduce soil-transmitted helminth (STH) infections in rural communities of Timor-Leste. The intervention included the construction or rehabilitation of household latrines to improve sanitation coverage, the installation of handwashing stations with soap and water to promote hygiene, and the provision of household water treatment systems to ensure safe drinking water. Hygiene education was also delivered to promote behavior change around handwashing and latrine use. This WASH intervention was delivered in combination with mass drug administration (MDA) of albendazole for deworming, and implemented through a cluster-randomized controlled trial, where villages were randomly assigned to either receive the integrated WASH and deworming intervention or continue with deworming alone. | The integrated WASH and deworming intervention did not have a significant effect on infection with Ascaris spp. (RR: 2.87, 95%CI: 0.66-12.48, P=0.159) or *Necator americanus* (RR: 0.99, 95%CI: 0.52-1.89, P=0.987), compared to deworming alone. At the last follow-up, open defecation was practiced by 66.1% (95% CI: 54.2-80.2) of respondents in the control arm versus 40.2% (95% CI: 25.3-52.6) of respondents in the intervention arm (P=0.005). No evidence was found that WASH intervention resulted in additional reductions in STH infections beyond that achieved with deworming alone over the 2-year trial period. The role of WASH on STH infections over a longer period of time and in the absence of deworming remains to be determined. |
| 7. | Gizaw, et al., 2019 | Ethiopia | Effects of water, sanitation and hygiene (WASH) education on childhood intestinal parasitic infections  in rural Dembiya, northwest Ethiopia: An uncontrolled before-and-after intervention study | To assess the impact of a WASH education program on household water, sanitation, hygiene practices, and the prevalence of intestinal parasitic infections among under-five children in rural Dembiya, northwest Ethiopia. | Location: Rural Dembiya district, northwest Ethiopia.  Participants: 225 children under 5 years  at baseline and 302 at endline survey (1-year follow-up). | Uncontrolled before-and-after  Intervention study | WASH education delivered weekly for 3 months, both after baseline and before endline: Community subgroups and schools, facilitated by teachers, students, religious leaders, and health workers were used.  Methods used include Role-play, group discussion, demonstrations, songs, and quizzes.  Participants were educated on personal hygiene, handwashing, safe water treatment, food safety,  waste management, transmission and prevention of parasitic infections. | *Infection prevalence:* Slight decrease from 25.8% at baseline to 23.8% at endline; not statistically significant (Prevalence Ratio: 0.92; 95%  CI: 0.62–1.38).  *WASH improvements:* Children with good hygiene rose from 1.3% to 34.4% (p < 0.05). Handwashing by caregivers improved from 24.4% to 68.2% (p < 0.001). Household water treatment increased from 7.6% to 47.0% (p < 0.001). Proper latrine usage increased from 32.0% to 49.0% (p < 0.05) |
| 8. | Pickering, et al., 2019 | Kenya | Effects of single and integrated water, sanitation, handwashing, and nutrition interventions on child soil-transmitted helminth  and giardia infections: A cluster-randomized controlled trial in rural Kenya | To evaluate the individual and combined effects of household-level water treatment, sanitation improvement, handwashing promotion, and nutrition supplementation on soil-transmitted helminths (STH) and Giardia infections in children over two years. | The study was carried out in rural western Kenya.  The trial participants were pregnant women and their children in 824 clusters.  Sample size was over 9,000 children enrolled in 6 treatment arms and 1 control group. | The study design used was a large cluster-randomized controlled trial (RCT).  The follow-up period was approximately 2 years.  Stool samples tested for *Ascaris lumbricoides*,  *Trichuris trichiura*, hookworm, and Giardia via microscopy and qPCR. | Children were exposed to one of the following arms:  Water treatment (W), Sanitation (S) – latrine upgrades, Handwashing (H) – provision of soap, WSH (combined water, sanitation, handwashing), Nutrition (N) – lipid-based nutrient supplements, WSH + N, and Control – no intervention  All households received annual deworming with albendazole to simulate standard MDA. | *A. lumbricoides* prevalence was significantly reduced in:  W arm: ~18% reduction, WSH arm: ~22% reduction, WSH + N arm: ~22% reduction.  Nutrition alone had no significant effect on *A. lumbricoides*, *T. trichiura*, or Giardia.  Giardia infection was not reduced by any intervention.  Hookworm and *T. trichiura* prevalence were too low for  meaningful analysis. |
| 9. | Ercumen, et al., 2019 | Bangladesh | Effects of water, sanitation, handwashing and nutritional interventions on soil-transmitted helminth infections in young  children: A cluster-randomized controlled trial in rural Bangladesh | To evaluate, in a setting with ongoing mass drug administration (MDA), the effects of water treatment, sanitation improvements, handwashing promotion, and nutritional supplementation – both single and in combination – on the prevalence and intensity of soil-transmitted helminth (STH) infections among children approximately 2.5 years after intervention rollout. | Index children (born to pregnant women enrolled around intervention start) aged ~30 months at follow-up.  Older children aged 2–12 years living in the same households or  compounds as index children (up to 2 additional children per  compound). | A cluster-randomized controlled trial, embedded within the larger WASH  Benefits trial. Multiple arms  compared the impacts of:  Water treatment (W: household-level), Handwashing (H: household-level), Sanitation upgrades (S: compound-level latrines,  potties, scoops),  Nutrition supplementation (N: index children only), Combinations of these into integrated WSH  (water, sanitation, handwashing)  and N+WSH interventions. | Water treatment: Chlorine tablets supplied to index households.  Sanitation: Upgraded latrines and hygiene hardware delivered to whole compound (all households in compound).  Handwashing: Stations with soapy water provided to index households.  Nutrition: Supplements given to index children.  Health promoter visits: ~6 times / month to support adherence, replenish consumables, and maintain hardware (index arms only). | *Hookworm:*  Water treatment alone led to a modest but sustained reduction in hookworm infection prevalence and egg counts across all enrolled children.  The strongest effect was observed among older children in the index households. Prevalence Ratio (PR): 0.59 (95% CI: 0.39–0.90) compared to control. No significant hookworm reduction among younger index children or children in other households  in the compound.  *T. trichiura and A. lumbricoides:*  Compound-level sanitation upgrades showed point estimates suggesting modest reductions in  *T. trichiura* and *A. lumbricoides*, but confidence intervals crossed the null, reflecting limited power and small subgroup sample sizes.  *Handwashing and Nutrition:*  Neither handwashing interventions nor nutritional supplementation yielded measurable reductions in any STH species, either alone or in combination arms—including integrated WSH or N+WSH combinations. |
| 10. | Chard, et al.,  2019 | Laos | Impact of a school-based water, sanitation, and hygiene intervention on school absence, diarrhea, respiratory infection, and soil-transmitted helminths:  Results from the WASH HELPS cluster-randomized  trial | To assess whether a comprehensive school-based WASH intervention reduces soil-transmitted helminth (STH) infections, school absenteeism, diarrhea, and respiratory infections among children in rural Laos. | 100 primary schools in Saravane Province, Lao People's Democratic Republic were selected for the study.  Thousands of schoolchildren enrolled in the study between 2014 and 2017. | A Cluster-randomized controlled trial (RCT) was used as the study design. It included two arms:  Intervention schools received the full  WASH package at baseline. Control schools received delayed  intervention (after data collection) | In the intervention arm, schools received:  Improved water supply infrastructure, Latrines (school sanitation facilities), Handwashing stations with soap and water, Drinking water filters, and Hygiene education and behavior promotion.  No direct household-level WASH interventions were included. | No statistically significant differences between intervention and control schools for: STH prevalence, Diarrhea, Respiratory infections, and School absence.  Despite high fidelity in delivering the intervention, health and attendance outcomes were not improved.  Suggests that school-based WASH interventions alone may be insufficient in settings where  household or environmental exposure persists. |
| 11. | Clarke, et al., 2018 | Timor-Leste | (S)WASH-D for Worms: A pilot study investigating the  differential impact of school-versus community-based  integrated control programs for soil-transmitted helminths | To pilot-test whether an integrated WASH plus deworming control program delivered at the community level could achieve greater reductions in soil-transmitted helminth (STH) infection prevalence among school-aged children than a school-based delivery model, and to assess feasibility and acceptability of the intervention approach in both settings | Six primary schools (3 intervention vs. 3 control) in a community where  open defecation was common.  Participants: School-aged children enrolled at these primary schools. | Design: A pilot, community and  school-based comparative trial.  Children were either subjected to school-based WASH and deworming, or an extended, community-wide WASH and deworming program.  Measurements taken at baseline and ~6 months follow-up included: deworming coverage, WASH  behavior, latrine usage, and STH prevalence / intensity. | All children received albendazole before latrine construction.  Intervention components included:  Construction of latrines at schools, WASH behavior change promotion, Implementation either at the school level or extended to the broader community.  Community-wide program included broader sanitation promotion beyond schoolchildren alone. | *Feasibility & Acceptability:*  High recruitment and participation rates, indicating strong acceptability of both intervention delivery methods among participants and communities.  School WASH implementation was successful, though sustaining latrine coverage community-wide remained challenging.  *Impact on Open Defecation:*  Open defecation among children decreased notably in both arms.  *STH Infection Outcomes:*  While the pilot was not adequately powered to detect statistically significant differences in infection prevalence changes, results provided preliminary evidence suggesting that community-wide programs may reduce STH prevalence more than school-only programs. |
| 12. | Campbell, et al., 2017 | Timor-Leste | Water, Sanitation and Hygiene (WASH) and environmental risk factors for soil-transmitted helminth intensity of infection in Timor-Leste, using real time PCR | To assess the association between household- and individual-level WASH and environmental factors and the intensity (not just presence) of *A. lumbricoides* and *Necator americanus* infections using quantitative real-time PCR (qPCR) in Timor-Leste. | 2,152 individuals from 24 rural communities in Manufahi District,  Timor-Leste | Cross-sectional baseline survey  using primary data. | No intervention was implemented. This was a baseline observational study.  Data collected included:  Stool samples analyzed via qPCR, Household WASH conditions: sanitation, water source, and handwashing facilities.  Environmental data: soil type, precipitation, vegetation cover.  Socioeconomic status and household density. | In adjusted models incorporating WASH, demographic and environmental variables were generally associated with infection intensity for both *N. americanus* and *Ascaris spp*. Precipitation was associated with increased risk of moderate-intensity (adjusted relative risk: 6.1, 95% CI: 1.9-19.3) and heavy-intensity (ARR: 6.6, 95% CI: 3.1-14.1) of *N. americanus* infection, as was sandy-loam soil around households (moderate-intensity ARR: 2.1, 95% CI: 1.0-4.3; heavy-intensity ARR: 2.7, 95% CI: 1.6-4.5; compared to no infection). For *Ascaris spp*, alkaline solid around the household was associated with reduced risk of moderate-intensity infection (ARR: 0.21, 95% CI: 0.09 - 0.51), and heavy intensity infection (ARR: 0.04, 95% CI: 0.01-0.25). Few WASH risk factors were significant. |
| 13. | Grimes, et al., 2016 | Ethiopia | School Water, Sanitation, and Hygiene, Soil-Transmitted Helminths, and Schistosomes: National Mapping in Ethiopia | To quantify the associations at a national level between school-level WASH conditions and infection intensity of soil‑transmitted helminths (STHs) and Schistosoma mansoni among schoolchildren, using data from national mapping surveys in Ethiopia | 80,475 schoolchildren, mostly aged 10–15 years. 1,645 Ethiopian schools surveyed during 2013–2014 national mapping of schistosomiasis and STH infections, with both WASH and parasitology data available. | An ecological, cross-sectional analysis at school level, linking WASH condition scores with arithmetic mean infection intensity (e.g. eggs per gram) in schools with at least one positive child. | Constructed quantitative WASH scores at each school:  Water score: Frequency and source of water collection (especially from freshwater bodies)  Sanitation score: Availability and condition of latrines (crowding, cleanliness, privacy)  Hygiene score: Presence of handwashing facilities, soap, water access | Better sanitation was associated with significantly lower *Ascaris lumbricoides* intensity (Kendall’s τb: -0.067, 95% CI: -0.11 to -0.023) and borderline significant lower hookworm intensity (Kendall’s τb: -0.039, 95% CI: -0.090 to 0.012, P = 0.067), and better hygiene was associated with significantly lower hookworm intensity (Kendall’s τb: -0.076, 95% CI: -0.13 to -0.020). However, no significant differences were observed when comparing sanitation and infection with *S. mansoni* or *T. trichiura,* or hygiene and infection with *A. lumbricoides* or *T. trichiura.* |
| 14. | Freeman, et al., 2015 | Kenya | Associations between school- and household-level water, sanitation and hygiene conditions and soil-transmitted helminth infection among Kenyan school children | To examine how water, sanitation, and hygiene (WASH) conditions at schools and households are associated with soil-transmitted helminth (STH) infections among Kenyan school-aged children. | Location: Rural Kenya  Participants: Over 8,000 schoolchildren across 69 schools | Cross-sectional observational study using baseline data from the Kenya National School-Based Deworming Programme | No intervention was applied in this study. The paper analyzed pre-existing conditions (WASH environment) and their association with infection prevalence. | Some school-level WASH conditions, such as having a reliable water source, were associated with lower STH prevalence.  Household sanitation access showed stronger associations with reduced risk than school WASH alone.  Results were mixed across different parasite species and WASH variables.  Results suggest that both household and school WASH improvements are needed to effectively reduce STH transmission. |
| 15. | Freeman, et al., 2013 | Kenya | The impact of a school-based hygiene, water quality and sanitation intervention on soil-transmitted helminth reinfection: A cluster-randomized trial | To evaluate whether a school-based WASH intervention reduces the reinfection rates of soil-transmitted helminths (STHs) after routine deworming in primary schoolchildren. | Forty government primary schools in Nyanza Province, Kenya were randomly selected and assigned to intervention or control arms. | Cluster-randomized controlled trial | Intervention arm: Schools received hygiene promotion, water treatment, and sanitation improvements.  Control arm: Schools received deworming  only (usual care).  *WASH Initiatives:*  Inclusion of handwashing facilities with water + soap in households, presence of school feeding programs, and access to improved drinking water sources. | *Ascaris lumbricoides:*  Significant reduction in reinfection (OR: 0.56, 95% CI: 0.31–1.00). Egg count intensity also significantly reduced (OR: 0.34).  No significant effect for *Trichuris trichiura*, hookworm, or *Schistosoma mansoni.*  Suggests that school WASH interventions may help reduce reinfection with *A. lumbricoides*, but not uniformly across all helminths. |
| 16. | Mascie‑Taylor, et al., 1999 | Bangladesh | A study of the cost effectiveness of selective health interventions for the control of intestinal parasites in rural Bangladesh | A study to determine the cost effectiveness of selective health interventions for the control of intestinal parasites in rural Bangladesh | Households with children aged 2 to 8 years in rural Bangladeshi villages | Randomized community trial over 18 months; four arms varying chemotherapy frequency and education | Albendazole (baseline, ± 6-month repeat for children), with or without health education on hygiene and sanitation | Chemotherapy-only every 18 months was most cost-effective; education-inclusive regimens were least efficient in reducing overall helminth burden, though they may have improved knowledge/behaviors |
