# Supplementary material for: Impact and cost-effectiveness of water, sanitation, hygiene, and health education interventions in the control and elimination of soil-transmitted helminthiasis: A scoping review
Source: PLoS Negl Trop Dis. 2026 Aug 25;20(8):e0014664. doi: 10.1371/journal.pntd.0014664 (PMC13529219; doi:10.1371/journal.pntd.0014664)
Supplement: Supplementary file 3 [file pntd.0014664.s003.docx]

**Review of the manuscript entitled “Impact and Cost-Effectiveness of Water, Sanitation, Hygiene, and Health Education Interventions in the Control and Elimination of Soil-Transmitted Helminthiasis: A Scoping Review" (PNTD-D-25-01970)**

The manuscript is well-written with almost no spelling or grammatical errors. The authors properly identified the study limitations and raised concern about the limited number of studies evaluating cost-effectiveness of implementing of these measures, long-term follow-up, and limited representation from certain regions, however, there are some points that need clarification which are listed below:

1. Why there was no line numbering included?
2. Page 3 at the end of the first, second and third paragraph, there is a sentence about reinfection and deworming, the authors should rearrange and combine these sentences.
3. The authors clearly identified the studied soil-transmitted helminthiasis (STH) as *Ascaris lumbricoides, Trichuris trichiura*, and hookworms. Why *Strongyloides stercoralis* was not included as STH?
4. The authors stated that in page 5 “The exclusion criteria eliminated studies that did not implement WASH interventions were not on STH”and in page 6 last line and page 7 first line that “Studies were excluded at this stage if they were clearly irrelevant or did not focus on WASH interventions in relation to soil-transmitted helminthiasis” so why was the study by Poague, et al.,2023 was included in the current review although it was done to explore the effect of improved access to water and sanitation services on *Schistosoma mansoni* prevalence. While the effect of WASH on *Schistosoma* can be studied alongside other helminths, in that particular research, *Schistosoma mansoni* is the only studied parasite and clearly it is not a soil transmitted helminth why was it included?
5. In page 5, second box from the right, WASH is written with extra ed (WASHed)
6. In the reference section, some of them have italic title while others are not, some with complete journal name while others with abbreviated name, please revise and correct according to the journal instructions to authors.
