# Supplementary material for: Impact and cost-effectiveness of water, sanitation, hygiene, and health education interventions in the control and elimination of soil-transmitted helminthiasis: A scoping review
Source: PLoS Negl Trop Dis. 2026 Aug 25;20(8):e0014664. doi: 10.1371/journal.pntd.0014664 (PMC13529219; doi:10.1371/journal.pntd.0014664)
Supplement: Supplementary file 4 [file pntd.0014664.s005.docx]

**Additional Editor Comments:**

"Timely manner" justifies broader strategies and no double screening, but this deviates from standard scoping review rigor, potentially introducing subjectivity.

**Author Response**:

We thank the reviewer for this clarification and apologize for the ambiguity created by the phrase “timely manner.” Our intention was not to justify reduced methodological rigor or broader strategies at the expense of standard scoping review practices. The review was conducted in accordance with the Arksey and O’Malley scoping review framework, using predefined eligibility criteria guided by the Population–Concept–Context (PCC) framework. Importantly, title/abstract and full-text screening were conducted independently by two reviewers, and any discrepancies were resolved through discussion and consensus, thereby minimizing subjectivity (see lines 186 – 191).

Question focuses on impact and cost-effectiveness of WASH and health education for STH control/elimination, but lacks specificity on outcomes (e.g., prevalence reduction, reinfection rates).

**Author Response**:

We thank the reviewer for this helpful comment. We agree that the original wording of the research question did not sufficiently specify the outcomes of interest. To address this, we have revised the research question to explicitly state the outcomes considered in this review. These include measures of infection (prevalence, intensity, and reinfection), costs, and cost-effectiveness (see lines 192-197).

Target populations limited to preschool/school-aged children and women of reproductive age, excluding general populations or adults, which may miss broader WASH-STH evidence; global scope and unrestricted time appropriate for scoping; exclusion of systematic reviews/meta-analyses without primary data reasonable, but could overlook synthesized evidence; PCC alignment noted but not explicitly mapped.

**Author Response**:

Regarding the target population, we chose to focus on preschool-aged children, school-aged children, and women of reproductive age because these groups are the primary targets of soil-transmitted helminth control programs and preventive chemotherapy strategies as reflected in WHO guidelines and NTD policies. We acknowledge that this may exclude some evidence from adult or general populations; however, these other populations are not the target of STH control programs. Inclusion of all population groups would have substantially expanded the scope of the review beyond its intended focus. We also chose to exclude systematic reviews and meta-analyses without primary data to ensure that the scoping review mapped original empirical evidence on impacts and economic outcomes.

Only PubMed and Google Scholar used, limiting comprehensiveness—missing specialized databases like Embase, Scopus, or Global Health; grey literature "reviewed" but no systematic search method described (e.g., specific repositories or hand-searching protocols).

**Author Response**:

PubMed was selected as the primary biomedical database because it provides comprehensive coverage of peer-reviewed literature on soil-transmitted helminthiasis, WASH interventions, and public health outcomes. Google Scholar was used as a complementary source to capture literature spanning public health, environmental health, economics, and implementation research, as well as to identify relevant articles that may not be indexed in PubMed. While we acknowledge that specialized databases such as Embase, Scopus, or Global Health could identify additional records, expanding database coverage could have substantially increased the volume of retrieved literature without necessarily improving relevance to the review’s focused population and concept. Grey literature search was performed by manually searching organizational websites, repositories, and reference lists, see lines 211-212.


Keywords comprehensive for WASH, STH species, and interventions, but OR/AND structure may yield overly broad results; inclusion of "mass drug administration" relevant for context but not core to WASH focus; no MeSH terms or synonyms for completeness (e.g., "latrine" for sanitation).

**Author Response**:

The keyword structure and term selection were intentionally designed to be broad in order to comprehensively capture literature spanning WASH interventions, soil-transmitted helminth species and different programmatic contexts. While this OR/AND structure may have yielded a large number of records, subsequent screening using predefined eligibility criteria ensured that only relevant studies were included in the review. The inclusion of “mass drug administration” in the search strategy was deliberate and reflects the aim of the review, which focuses on WASH and health education interventions that complement deworming programs. Including this term ensured the retrieval of studies where WASH components were evaluated alongside standard STH control strategies. With respect to additional synonyms (e.g., MeSH terms or terms such as “latrine”), we acknowledge that the search strategy did not exhaustively include all possible indexed terms. However, Google Scholar does not support MeSH indexing, and PubMed’s automatic term mapping was relied upon to capture relevant synonyms and related concepts.


Single screening for titles/abstracts and full texts increases risk of errors/inconsistencies; Mendeley used appropriately; eligibility tied to PCC, but no mention of pilot testing or inter-rater agreement; flow diagram (Figure 1) referenced but numbers/reasons for exclusions not summarized here.

**Author Response**:

We thank the reviewer for this comment. We would like to clarify that title/abstract and full-text screening were in fact conducted independently by two reviewers using predefined eligibility criteria guided by the Population–Concept–Context (PCC) framework. Discrepancies were resolved through discussion and consensus among the review team. The description of duplicate screening was inadvertently omitted from the original draft, and we apologize for this oversight. The Methods section has now been revised to explicitly state that double screening was undertaken (See lines 187 – 192).

Regarding pilot testing and inter-rater agreement, we confirm that formal pilot screening and quantitative inter-rater reliability statistics were not conducted. Given the clearly defined inclusion and exclusion criteria and the scoping nature of the review, screening decisions were harmonized through team discussions rather than formal reliability assessment. This has now been clarified in the revised manuscript. This has also been noted as an additional limitation of the study (See lines 433 – 438).

The PRISMA flow diagram presents the number of records identified, screened, included and excluded at each stage, along with reasons for full-text exclusions, in accordance with PRISMA-ScR guidance. To avoid redundancy, these details were not repeated in the main text; however, we agree that the flow diagram serves as the primary source of this information and have ensured that it is clearly referenced and complete.


**Journal Requirements:**

If the reviewer comments include a recommendation to cite specific previously published works, please review and evaluate these publications to determine whether they are relevant and should be cited. There is no requirement to cite these works unless the editor has indicated otherwise.

1) Please upload all main figures as separate Figure files in .tif or .eps format. For more information about how to convert and format your figure files please see our guidelines: 
https://journals.plos.org/plosntds/s/figures

2) We have noticed that you have uploaded Supporting Information files, but you have not included a list of legends. Please add a full list of legends for your Supporting Information files after the references list.

3) We note that your Data Availability Statement is currently as follows: "All relevant data are included within the paper.". Please confirm at this time whether or not your submission contains all raw data required to replicate the results of your study. Authors must share the “minimal data set” for their submission. PLOS defines the minimal data set to consist of the data required to replicate all study findings reported in the article, as well as related metadata and methods (https://journals.plos.org/plosone/s/data-availability#loc-minimal-data-set-definition).
For example, authors should submit the following data: 
1) The values behind the means, standard deviations and other measures reported;
2) The values used to build graphs;
3) The points extracted from images for analysis..
Authors do not need to submit their entire data set if only a portion of the data was used in the reported study.
If your submission does not contain these data, please either upload them as Supporting Information files or deposit them to a stable, public repository and provide us with the relevant URLs, DOIs, or accession numbers. For a list of recommended repositories, please see https://journals.plos.org/plosone/s/recommended-repositories. 
If there are ethical or legal restrictions on sharing a de-identified data set, please explain them in detail (e.g., data contain potentially sensitive information, data are owned by a third-party organization, etc.) and who has imposed them (e.g., an ethics committee). Please also provide contact information for a data access committee, ethics committee, or other institutional body to which data requests may be sent. If data are owned by a third party, please indicate how others may request data access.

**Reviewers' Comments:**

Reviewer's Responses to Questions

**Key Review Criteria Required for Acceptance?**

As you describe the new analyses required for acceptance, please consider the following:

**Methods**

-Are the objectives of the study clearly articulated with a clear testable hypothesis stated?

-Is the study design appropriate to address the stated objectives?

-Is the population clearly described and appropriate for the hypothesis being tested?

-Is the sample size sufficient to ensure adequate power to address the hypothesis being tested?

-Were correct statistical analysis used to support conclusions?

-Are there concerns about ethical or regulatory requirements being met?

Reviewer #1: Pls provide the table as the supplemental file which will reduce the length of the volume of the article

**Author Response**:

To reduce the length of the main manuscript and improve readability, the table has been moved to the supplementary materials and is now provided as a supplemental file. See supplementary table S1.

Reviewer #2: (No Response)

Reviewer #3: (No Response)

**Results**

-Does the analysis presented match the analysis plan?

-Are the results clearly and completely presented?

-Are the figures (Tables, Images) of sufficient quality for clarity?

Reviewer #1: Please mention about the name of the parasites found in the included articles.
Pls mention the name of the anthelmintics used in the MDA of the included studies
Provide the results of the MDA and MDA+WASH stated in the different articles
Nothing mentioned about the cost MDAs or MDAs+WASH; Nothing is free.

**Author Response**:

We thank the reviewer for this comment. Table 1 summarises the interventions deployed (including drugs used), and the key results from each included paper. The majority of the papers did not report on the costs. Further, we have revised the manuscript text to explicitly summarize the STH species evaluated across included studies, noting that *Ascaris lumbricoides, Trichuris trichiura,* and hookworm species were most commonly assessed (see lines 272-274).

Reviewer #2: (No Response)

Reviewer #3: (No Response)

**Conclusions**

-Are the conclusions supported by the data presented?

-Are the limitations of analysis clearly described?

-Do the authors discuss how these data can be helpful to advance our understanding of the topic under study?

-Is public health relevance addressed?

Reviewer #1: (No Response)

Reviewer #2: (No Response)

Reviewer #3: (No Response)

**Editorial and Data Presentation Modifications?**

Use this section for editorial suggestions as well as relatively minor modifications of existing data that would enhance clarity. If the only modifications needed are minor and/or editorial, you may wish to recommend “Minor Revision” or “Accept”.

Reviewer #1: (No Response)

Reviewer #2: (No Response)

Reviewer #3: Dear Editor,
Re: Reviewer Comments:
Thank you for giving me the privilege to review manuscript, “Impact and Cost-Effectiveness of Water, Sanitation, Hygiene, and Health Education Interventions in the Control and Elimination of Soil-Transmitted Helminthiasis: A Scoping Review”
Find below my comments on the MS.
1. Abstract
• The background section is inadequate and does not sufficiently contextualize the study or clearly articulate the rationale for the scoping review. The reader is not adequately oriented to the existing evidence gap.

**Author Response**:

Thank you for this comment. We have revised the Abstract to strengthen the Background by more clearly contextualizing the persistent challenge of STH control despite mass drug administration, the role of WASH interventions, and the existing evidence gap, particularly regarding the cost-effectiveness of integrated WASH and MDA interventions (see lines 18-26).

• The sentence under Methods (“This scoping review aimed to systematically map and summarize the impact and cost-effectiveness of WASH evidence for STH control and elimination efforts.”) is more appropriate in the Background, as it states the study objective rather than a methodological procedure.

**Author Response**:

Thank you for this comment. We have moved the sentence (“This scoping review aimed to…”) from the Methods to the Background section of the Abstract. The Methods section now focuses solely on the scoping review approach and the data sources (see lines 24-26).

2. Discussion
• The discussion is underdeveloped and does not meet current standards for scoping reviews. The authors fail to situate their findings within existing literature, and the absence of citations limits interpretability and undermines the contribution of the review. In line with PRISMA-ScR guidance, the discussion should explicitly relate the mapped evidence to prior studies, highlight consistencies or gaps, and clarify how the findings advance the field.

**Author Response**:

We have substantially revised and expanded the discussion section to align with PRISMA-ScR guidance and standards for scoping reviews.

Specifically, we have revised the discussion to

i.) Explicitly situate our findings within the existing literature on WASH and soil-transmitted helminth (STH) control.
ii.) Clearly articulated inconsistencies and evidence gaps that were identified through the scoping review process.

The revised Discussion now integrates findings from recent intervention and observational studies, including quasi-experimental evidence on integrated WASH education deworming (Nachaiwieng et al., 2024), population-based observational data from the Geshiyaro project in Ethiopia (Phillips et al., 2022), and randomized trial evidence from the WASH for WORMS study (Nery et al., 2019). These studies are used to put into context our review findings, highlight varying effects across parasite species and settings, and emphasize the importance of community-level WASH coverage.

In addition, we have explicitly identified and discussed major gaps in the evidence base, particularly the lack of robust and contemporary cost and cost-effectiveness evaluations of integrated WASH and deworming interventions. A dedicated limitations paragraph has been added to acknowledge this constraint and to clarify its implications for policy and resource-allocation decisions (See lines 428 – 433)

3. Specific Statements for Clarification
• “However, behavioral improvements alone were insufficient without supporting infrastructure, and their effects on other helminths were inconsistent”: It is unclear whether “other helminths” refers specifically to other STH species or helminths more broadly. This distinction is important, as not all helminths are primarily driven by hygiene and sanitation conditions. The authors should clarify the scope of this statement.

**Author Response**:

The discussion has been refined to remove ambiguity regarding helminth species by explicitly specifying that statements referring to heterogeneous effects apply to soil-transmitted helminth species, including *Ascaris lumbricoides, Trichuris trichiura, and* hookworm, rather than helminths more broadly (See line 374).

• “At the household level, integrated WASH packages… led to an approximate 22% reduction in A. lumbricoides prevalence”: This quantitative statement is problematic in a scoping review context. The manuscript does not explain whether this figure comes from a single study, a pooled estimate, or descriptive synthesis. Note that, scoping reviews should avoid meta-analytic or effect-size language unless a formal quantitative synthesis is conducted. The source of this estimate should be clarified, and the wording revised to reflect descriptive reporting.

**Author Response**:

We thank the reviewer for this important clarification and agree that the original wording was not appropriate for a scoping review. The intent was to descriptively summarize patterns reported across individual studies rather than to present a pooled or meta-analytic effect estimate. To address this, we have revised the text to remove quantitative effect-size language and replaced it with descriptive wording that reflects variation in findings across studies (See lines 313 – 314).

**Summary and General Comments**

Use this section to provide overall comments, discuss strengths/weaknesses of the study, novelty, significance, general execution and scholarship. You may also include additional comments for the author, including concerns about dual publication, research ethics, or publication ethics. If requesting major revision, please articulate the new experiments that are needed.

Reviewer #1: The topic is interesting but requires substantial modifications before acceptance
Abstract and Author summary
Pls provide the results in this sections

**Author Response**:

The Abstract and Author Summary have been revised to explicitly include key results of the scoping review. These sections now summarize the main findings regarding the impact of integrated WASH and deworming interventions on soil-transmitted helminth outcomes.

Introductions
WASH: stands only for water, sanitation, and hygiene; so put 'WASH' before 'and'
Pls provide a paragraph on the impacts of the STFs

**Author Response**:

In this manuscript, our definition for WASH is water, sanitation, hygiene, and health education. We have used this abbreviation consistently throughout the text. The impacts of soil-transmitted helminth infections are already described in the Introduction, where we outline their effects on public health, nutrition, cognitive development, educational attainment and economic productivity, particularly among school-aged children (See lines 134 – 137).


Discussion
Very poorly discussed. Please discuss your findings point by point

**Author Response**:

We have substantially revised and expanded the discussion section to align with PRISMA-ScR guidance and standards for scoping reviews.

Specifically, we have revised the discussion to

i.) Explicitly situate our findings within the existing literature on WASH and soil-transmitted helminth (STH) control.
ii.) Clearly articulated inconsistencies and evidence gaps identified through the scoping review process.

The revised Discussion now integrates findings from recent intervention and observational studies, including quasi-experimental evidence on integrated WASH education deworming (Nachaiwieng et al., 2024), population-based observational data from the Geshiyaro project in Ethiopia (Phillips et al., 2022), and randomized trial evidence from the WASH for WORMS study (Nery et al., 2019). These studies are used to put into context our review findings, highlight varying effects across parasite species and settings, and emphasize the importance of community-level WASH coverage.

In addition, we have explicitly identified and discussed major gaps in the evidence base, particularly the lack of robust and contemporary cost and cost-effectiveness evaluations of integrated WASH and deworming interventions. A dedicated limitations paragraph has been added to acknowledge this constraint and to clarify its implications for policy and resource-allocation decisions.

Reviewer #2: (No Response)

Reviewer #3: The manuscript addresses an important topic, but the current version requires some revisions. Key issues include insufficient contextualization in the abstract and discussion, lack of citations to situate findings within the literature (discussion section), ambiguous statements regarding helminth effects, and inappropriate use of quantitative effect language in a scoping review. Addressing these issues will enhance the rigor, clarity, and contribution of the manuscript.

**Author Response**:

We have revised the manuscript to address all points raised. Specifically, the discussion has been substantially expanded with additional citations to situate our findings within the existing literature and to highlight consistencies and gaps. Ambiguous statements regarding helminth effects have been clarified to explicitly refer to soil-transmitted helminth species, and quantitative effect-size language has been revised to reflect descriptive synthesis consistent with scoping review methodology. We believe these revisions substantially improve the rigor, clarity, and contribution of the manuscript.

PLOS authors have the option to publish the peer review history of their article ([what does this mean?](https://journals.plos.org/plosntds/s/editorial-and-peer-review-process#loc-peer-review-history)). If published, this will include your full peer review and any attached files.

If you choose “no”, your identity will remain anonymous but your review may still be made public.

**Do you want your identity to be public for this peer review?** For information about this choice, including consent withdrawal, please see our [Privacy Policy](https://www.plos.org/privacy-policy).

Reviewer #1: **Yes:** Anisuzzaman

Reviewer #2: No

Reviewer #3: No

[NOTE: If reviewer comments were submitted as an attachment file, they will be attached to this email and accessible via the submission site. Please log into your account, locate the manuscript record, and check for the action link "View Attachments". If this link does not appear, there are no attachment files.]

**Figure resubmission:**

While revising your submission, we strongly recommend that you use PLOS’s NAAS tool (https://ngplosjournals.pagemajik.ai/artanalysis) to test your figure files. NAAS can convert your figure files to the TIFF file type and meet basic requirements (such as print size, resolution), or provide you with a report on issues that do not meet our requirements and that NAAS cannot fix.

After uploading your figures to PLOS’s NAAS tool - https://ngplosjournals.pagemajik.ai/artanalysis, NAAS will process the files provided and display the results in the "Uploaded Files" section of the page as the processing is complete. If the uploaded figures meet our requirements (or NAAS is able to fix the files to meet our requirements), the figure will be marked as "fixed" above. If NAAS is unable to fix the files, a red "failed" label will appear above. When NAAS has confirmed that the figure files meet our requirements, please download the file via the download option, and include these NAAS processed figure files when submitting your revised manuscript.

**Reproducibility:**

To enhance the reproducibility of your results, we recommend that authors of applicable studies deposit laboratory protocols in protocols.io, where a protocol can be assigned its own identifier (DOI) such that it can be cited independently in the future. Additionally, PLOS ONE offers an option to publish peer-reviewed clinical study protocols. Read more information on sharing protocols at https://plos.org/protocols?utm_medium=editorial-email&utm_source=authorletters&utm_campaign=protocols

*In compliance with data protection regulations, you may request that we remove your personal registration details at any time.*  [*(Remove my information/details)*](https://www.editorialmanager.com/pntd/login.asp?a=r)*. Please contact the publication office if you have any questions.*

**Review of the manuscript entitled “Impact and Cost-Effectiveness of Water, Sanitation, Hygiene, and Health Education Interventions in the Control and Elimination of Soil-Transmitted Helminthiasis: A Scoping Review" (PNTD-D-25-01970)**

The manuscript is well-written with almost no spelling or grammatical errors. The authors properly identified the study limitations and raised concern about the limited number of studies evaluating cost-effectiveness of implementing of these measures, long-term follow-up, and limited representation from certain regions, however, there are some points that need clarification which are listed below:

1. Why there was no line numbering included?

**Author Response**:

The manuscript has been updated to include line numbering.

2. Page 3 at the end of the first, second and third paragraph, there is a sentence about reinfection and deworming, the authors should rearrange and combine these sentences.

**Author Response**:

We thank the reviewer for this suggestion. We have carefully considered the comment and would like to clarify that the references to reinfection and deworming at the end of each paragraph were intentionally retained. Each paragraph addresses a distinct aspect of soil-transmitted helminth control, and the concluding sentences serve to reinforce the relevance of reinfection within each specific context. We believe that combining these statements into a single paragraph would reduce the clarity of the concept by weakening the link between reinfection dynamics and the focus of each section.

3. The authors clearly identified the studied soil-transmitted helminthiasis (STH) as *Ascaris lumbricoides, Trichuris trichiura*, and hookworms. Why *Strongyloides stercoralis* was not included as STH?

**Author Response**:

While *Strongyloides stercoralis* is sometimes grouped under soil-transmitted helminths, this scoping review adopted the World Health Organization preventive chemotherapy framework, which primarily targets *Ascaris lumbricoides, Trichuris trichiura,* and hookworm species.

4. The authors stated that in page 5 “The exclusion criteria eliminated studies that did not implement WASH interventions were not on STH”and in page 6 last line and page 7 first line that “Studies were excluded at this stage if they were clearly irrelevant or did not focus on WASH interventions in relation to soil-transmitted helminthiasis” so why was the study by Poague, et al.,2023 was included in the current review although it was done to explore the effect of improved access to water and sanitation services on *Schistosoma mansoni* prevalence. While the effect of WASH on *Schistosoma* can be studied alongside other helminths, in that particular research, *Schistosoma mansoni* is the only studied parasite and clearly it is not a soil transmitted helminth why was it included?

**Author Response**:

We acknowledge that *Schistosoma mansoni* is not classified as a soil-transmitted helminth, and the inclusion of Poague et al. (2023), which focused exclusively on schistosomiasis outcomes, was not consistent with our eligibility criteria. The manuscript has been revised to remove this study from the included articles. See supplementary table 1.

5. In page 5, second box from the right, WASH is written with extra ed (WASHed)

**Author Response**:

The manuscript has been revised to replace the previously used terminology with “WASH” throughout, ensuring consistency and alignment with standard usage in the literature. In this study, WASH stands for water, sanitation, hygiene, and health education.

6. In the reference section, some of them have italic title while others are not, some with complete journal name while others with abbreviated name, please revise and correct according to the journal instructions to authors.

**Author Response**:

The reference list has been revised and standardized to ensure consistent formatting throughout, including journal title presentation and use of italics, in accordance with the PLOS Neglected Tropical Diseases instructions for Authors.
