# Supplementary material for: Impact and cost-effectiveness of water, sanitation, hygiene, and health education interventions in the control and elimination of soil-transmitted helminthiasis: A scoping review
Source: PLoS Negl Trop Dis. 2026 Aug 25;20(8):e0014664. doi: 10.1371/journal.pntd.0014664 (PMC13529219; doi:10.1371/journal.pntd.0014664)
Supplement: Supplementary file 5 [file pntd.0014664.s006.docx]

**Reviewer #4: The study objectives are clearly articulated and address a relevant and timely topic, namely the impact and cost-effectiveness of WASH interventions for the control of soil-transmitted helminthiasis. The use of the Arksey and O’Malley framework alongside PRISMA-ScR guidelines is appropriate for a scoping review and aligns with current methodological standards. However, there are some limitations that reduce the overall rigor of the methodology. The search strategy appears somewhat limited, as it relies primarily on PubMed and Google Scholar, which may restrict the comprehensiveness of the evidence base. In addition, the grey literature search is not described in sufficient detail to ensure reproducibility. While the authors clarified that screening was conducted independently, the absence of inter-rater reliability assessment and pilot testing reduces confidence in the consistency of study selection. The population focus is generally appropriate, although excluding broader adult or community-level populations may limit the scope of the findings. The study design is suitable, but greater transparency and comprehensiveness are needed.**

***Author Response***

*Thank you for this thoughtful feedback. We appreciate the recognition of the study’s relevance and methodological foundation, and we have addressed the concerns raised to improve the transparency and rigor of the review.*

*First, regarding the search strategy, we acknowledge that relying on PubMed and Google Scholar may appear limited. These databases were selected because they provide broad coverage of biomedical and interdisciplinary literature, including public health, WASH, and economic evaluations. In addition, Google Scholar was specifically included to capture cross-sectoral and non-indexed studies. To further strengthen transparency, we have clarified this rationale in the revised manuscript. See lines 274 – 277.*

*Second, we agree that the description of the grey literature search required more detail. We have now expanded this section to specify the types of sources consulted, including organizational websites (e.g., WHO), conference proceedings, and reference list screening. This has been revised to improve reproducibility. See lines 240 – 244.*

*Third, regarding inter-rater reliability and pilot testing, we acknowledge that these were not formally conducted. However, study screening was performed independently by two reviewers using predefined PCC-based criteria, and discrepancies were resolved through discussion and consensus. We have acknowledged this as a limitation in the Discussion. See lines 580 – 588.*

*Finally, regarding the population scope, the focus on preschool and school-aged children and women of reproductive age was intentional, as these are the primary target groups for STH control programs and WHO guidelines. However, we acknowledge that excluding broader community populations may limit the generalizability of findings, and we have now highlighted this more clearly as a limitation in the Discussion. See lines 580 – 588.*

*We believe these revisions improve the clarity, transparency, and methodological rigor of the manuscript.*

**Reviewer #4: The results are generally consistent with the stated objectives and follow the described analytical approach. The findings are clearly presented and logically organized, with appropriate descriptive synthesis consistent with scoping review methodology. The manuscript does a good job of categorizing studies by design and geographic distribution, and it highlights important gaps in the literature, particularly the limited evidence on cost-effectiveness. However, the reporting of intervention details is somewhat limited, especially regarding variability in mass drug administration regimens and the specific WASH components implemented. The discussion of cost-related findings is relatively minimal and would benefit from further elaboration. In some instances, the results remain largely descriptive without sufficient comparison or synthesis across studies. The figures and tables are generally appropriate and clear, although additional comparative summaries could improve clarity and interpretation.**

***Author Response***

*Thank you for your detailed feedback. We appreciate your positive assessment of the organization of the results, and we have substantially strengthened the results section to improve the depth of reporting and synthesis.*

*First, regarding the level of intervention detail, we have revised the Results section and Supplementary Table S1 to more explicitly describe variation in both mass drug administration (MDA) regimens and WASH components across studies. The included studies demonstrate considerable diversity in MDA frequency, delivery platforms, and coverage levels. Similarly, WASH interventions ranged from education-only approaches to comprehensive packages incorporating water treatment, sanitation infrastructure, handwashing facilities, and behavior change promotion. These distinctions are now more clearly articulated in the Results. See section 3.2.*

*Second, we have expanded the synthesis of findings across studies. Rather than presenting results primarily descriptively, we now draw clearer comparisons across intervention types, study designs, and epidemiological outcomes.*

*Third, we have strengthened the discussion of cost-related findings. Although only one study conducted a formal cost-effectiveness analysis, we now provide a more detailed interpretation of its findings in relation to the broader evidence base. We also discuss how the absence of economic evaluations across diverse intervention types limits the ability to compare the relative value of integrated WASH strategies vs MDA alone.*

*Finally, we have improved the clarity of results presentation by incorporating additional comparative summaries, highlighting patterns across geographical settings, intervention intensity, and species-specific outcomes. These revisions enhance the interpretability of the findings while remaining consistent with the objectives of a scoping review.*

*We believe these changes substantially improve the analytical depth and overall contribution of the manuscript.*

**Reviewer #4: The conclusions are generally supported by the data presented and appropriately emphasize the potential benefits of integrating WASH interventions with mass drug administration. The manuscript effectively highlights key evidence gaps, particularly the lack of robust economic evaluations. However, the conclusions may slightly overstate the strength of the evidence, given the heterogeneity of included studies and the limited number of cost-effectiveness analyses. The policy implications could be framed more cautiously in light of these limitations. Nonetheless, the public health relevance of the study is strong and clearly articulated.**

***Author Response***

*We have revised the Conclusion section to adopt a more cautious interpretation of the evidence. We have also refined the framing of policy implications to acknowledge the limitations of the current evidence base, particularly the limited number of cost-effectiveness analyses. The revised conclusion now emphasizes the potential of integrated approaches while highlighting the need for further high-quality and context-specific research to guide implementation and investment decisions.*

**Reviewer #4: The manuscript would benefit from improved clarity and consistency in terminology, particularly in distinguishing among different soil-transmitted helminth species. The reporting of intervention components should be strengthened to better differentiate between WASH and mass drug administration elements. The description of the grey literature search strategy should be expanded to enhance reproducibility. All supplementary materials, including tables and the PRISMA flow diagram, should be clearly referenced and labeled within the text. The manuscript is generally well written in English, although minor editing would further improve clarity.**

***Author Response***

*We have carefully revised the manuscript to improve clarity, consistency, and transparency, as suggested.*

*First, we have reviewed and standardized the terminology for soil-transmitted helminth species throughout the manuscript. Where appropriate, we now consistently distinguish among Ascaris lumbricoides, Trichuris trichiura, and hookworm species to improve clarity.*

*Second, we have strengthened the reporting of intervention components throughout the text to more clearly differentiate between WASH and mass drug administration (MDA) elements.*

*Third, we have expanded the description of the grey literature search strategy to enhance reproducibility. The revised Methods section now provides additional detail on the sources consulted, including organizational websites, institutional repositories, and reference list screening. See section 2.3.*

*Fourth, we have ensured that all supplementary materials, including tables and the PRISMA flow diagram, are clearly referenced and appropriately labeled within the main text.*

*Finally, the manuscript has undergone additional language editing to improve clarity and readability.*

*We believe these revisions have improved the overall clarity and consistency of the manuscript.*

**Reviewer #3: The authors have made substantial improvement to the manuscript. However, I still think the discussion is shallow and needs additional inputs.**

***Author Response***

*In response to this comment, we have reworked the Discussion to provide a clearer interpretation of the results. Specifically, we have added additional interpretation to explain variation in findings across studies, including the influence of contextual factors such as baseline transmission intensity, environmental contamination, and intervention coverage.*

*We have also introduced a dedicated paragraph on policy and programmatic implications, highlighting the importance of integrating WASH interventions with MDA for sustainable STH control, as well as the challenges posed by limited evidence on cost-effectiveness for decision-making in resource-constrained settings.*

**Correct the spelling of "discrepancies" on line 209**

***Author Response***

*We have corrected the spelling. See line 209.*

**Check that the referencing format is correct and is applied throughout the text.**

***Author Response***

*All the references have been updated with a consistent format throughout the text.*

**Note that this observation, “Freeman et al., 2013 demonstrated a 44% reduction in reinfection risk (OR: 0.56, 95% CI: 0.31–1.00)” is not considered statistically significant. - Lines 315 to 316.**

***Author Response***

*Thank you for this important clarification regarding the interpretation of the reported estimate. We agree that the confidence interval (95% CI: 0.31-1.00) indicates that the result is not statistically significant.*

*In the revised manuscript, the Results section has been substantially updated, and the specific statement is no longer present.*

**Revise line 318 to:
“However, no significant effects were observed for T. trichiura or hookworm. Similarly, a community-wide trial in Timor-Leste (WASH for WORMS) achieved a 25.9 percentage-point reduction in open defecation (66.1% to 40.2%), but did not significantly reduce STH prevalence compared with MDA alone.”**

***Author Response***

*In the revised manuscript, the Results section has been substantially reorganized and expanded to improve overall clarity. As part of these revisions, the specific sentence previously appearing on line 226 has been incorporated into a broader, more integrated discussion of species-specific outcomes and community-level intervention effects.*

**Reviewer #4: This manuscript addresses an important and policy-relevant topic in the control and elimination of neglected tropical diseases. The integration of WASH interventions with deworming programs is of significant interest to researchers, practitioners, and policymakers. The study is strengthened by its use of an appropriate scoping review framework and its clear identification of key evidence gaps, particularly in relation to cost-effectiveness. The discussion has improved and provides useful context for the findings. However, the manuscript is limited by a relatively narrow search strategy, insufficient detail in the grey literature search, and a lack of depth in the synthesis of economic evidence. Some sections remain overly descriptive rather than analytical. The manuscript has merit but requires moderate to substantial revisions to improve methodological transparency, depth of analysis, and clarity before it can be considered suitable for publication.**

***Author Response***

*We appreciate the reviewer’s recognition of the study’s relevance, the use of an appropriate scoping review framework, and the identification of key evidence gaps.*

*In response, we have undertaken substantial revisions to improve the manuscript's methodological transparency, depth of analysis, and overall clarity. Specifically, the search strategy has been further clarified, including a more detailed justification for database selection. See lines 274 – 277. In addition, the description of the grey literature search has been expanded to improve reproducibility, with clearer specification of sources and search approaches. See lines 240 – 244.*

*We have also strengthened the analytical depth of the manuscript, particularly in the Results and Discussion sections. The revised version now includes more explicit comparisons across studies, including differences in intervention types, implementation levels, and species-specific outcomes. Furthermore, the Discussion has been expanded to provide deeper interpretation of findings, including factors influencing variability in outcomes and their implications for policy and practice.*

*With respect to economic evidence, we have enhanced the synthesis and interpretation of cost-related findings, while explicitly acknowledging the limited availability of cost-effectiveness studies as a key gap in the literature.*

*We believe these revisions comprehensively address the concerns raised and significantly strengthen the manuscript.*
