# Supplementary material for: Impact and cost-effectiveness of water, sanitation, hygiene, and health education interventions in the control and elimination of soil-transmitted helminthiasis: A scoping review
Source: PLoS Negl Trop Dis. 2026 Aug 25;20(8):e0014664. doi: 10.1371/journal.pntd.0014664 (PMC13529219; doi:10.1371/journal.pntd.0014664)
Supplement: Supplementary file 6 [file pntd.0014664.s007.docx]

**Review Comments:**

Reviewer #5: Revision 2 represents a clear improvement over the previous version. The authors articulate the objectives of this scoping review with appropriate clarity. Their aim is to map and summarize the evidence on the impact and cost-effectiveness of water, sanitation, hygiene, and health education interventions for soil-transmitted helminthiasis. A formal hypothesis is not expected for a scoping review, and the study design follows the well-established Arksey and O'Malley framework alongside PRISMA ScR guidelines, which is entirely appropriate. The target populations, including preschool children, school-aged children, and women of reproductive age, are clearly described and relevant given that these groups experience the highest infection burden. The sample size of sixteen studies is sufficient to identify key evidence gaps and patterns across the literature. The descriptive synthesis is correct for this study type, and no inappropriate inferential statistics are applied. Ethical requirements are satisfied because the review synthesizes previously published data only. Two minor issues require correction. First, the grey literature search description lacks sufficient detail for reproducibility; the authors mention manual searches of organizational websites and conference proceedings but do not specify which organizations, which conferences, or the dates of those searches. Second, the reference list contains formatting inconsistencies, including missing journal names in several entries and mixed use of abbreviated versus full journal titles. Both issues can be addressed with minor revisions.

**AUTHOR RESPONSE**

**We thank the reviewer for their positive evaluation of our scoping review and for highlighting these important areas for improvement. We have addressed both minor issues in the updated version of the manuscript as detailed below:**

**- We have revised Section 2.3 (Databases searched) to explicitly list the specific organizational websites, institutional repositories, and conference proceedings that were manually searched.**

**- We have revised the reference list to correct all formatting inconsistencies.**

Reviewer #6: -Sorta.
The aims of the study are clearly stated, and seek to map and summarize the evidence on the impact and cost-effectiveness of WASH interventions for the control and elimination of soil-transmitted helminthiasis (STH). The research issue is well stated and appropriate to the approach of a scoping review. But there is no explicit tested hypothesis. This is understandable as scoping reviews are mainly exploratory and their goal is to map the existing evidence rather than to test a hypothesis formulated a priori. However, the authors may improve conceptual clarity by explicitly addressing the expected association between integrated WASH interventions and STH outcomes.

**AUTHOR RESPONSE**

**We agree with the reviewer that establishing a clear conceptual expectation enhances the review's clarity and depth, even in the absence of a formal statistical hypothesis. To address this, we have added a paragraph at the end of the introduction outlining the expected associations between integrated WASH interventions and STH outcomes. Specifically, we have clarified that MDA reduces immediate individual worm burdens, while WASH infrastructure and behavioural interventions are expected to complement this by interrupting environmental transmission pathways, thereby leading to sustained reductions in reinfection and prevalence compared with MDA alone. (See lines 158 – 168)**

-Yes.
The stated purpose of this review is to identify, map and synthesize available data on the impact and cost-effectiveness of WASH interventions, and therefore the adoption of a scoping review approach following the Arksey and O’Malley framework and PRISMA-ScR standards is suitable. The study design is appropriate for identification of knowledge gaps and provides a broad perspective of the literature. However, the scope of the review is somewhat constrained by the narrow database selection and the not detailed enough grey literature search approach.

**AUTHOR RESPONSE**

**We appreciate the reviewer’s feedback on the scope and comprehensiveness of our search strategy. While PubMed and Google Scholar provided excellent coverage of the intersection between public health interventions and economic evaluations, we recognise that using only two primary databases is a limitation. We have explicitly added this limitation to the discussion section (See lines 449-471). We have also highlighted how our rigorous backward citation tracking served as a safety net to capture articles not indexed in the primary searches. Additionally, we have expounded on the details of the grey literature search as indicated in section 2.3.**

-Yes.
The target populations are well defined, include preschool-aged children, school-aged children and women of reproductive age, and reflect the main categories addressed by WHO-approved STH management programmes. These populations are extremely relevant for the purposes of the review. Nonetheless, limiting the analysis to smaller community populations may hinder the generalizability of findings, especially for the assessment of community-wide WASH interventions and transmission dynamics.

**AUTHOR RESPONSE**

**We agree that while targeting preschool children, school-aged children, and women of reproductive age aligns directly with WHO’s high-risk stratification for preventive chemotherapy, it can create a blind spot regarding broader community-wide parasite transmission dynamics. We have added text in section 3.4 (School vs Community-Level interventions) emphasising that shifting environmental transmission dynamics requires looking beyond high-risk subgroups to the broader community. See lines 342-352.**

**In the discussion section, we have explicitly stated that our focus on these key demographic groups may limit the generalizability of the findings to macro-level community transmission, where adult populations not captured by the targeted metrics can still sustain environmental reservoirs of infection, see lines 455-465.**

-Unable to evaluate properly / Not applicable.
As this is a scoping study, traditional considerations of sample size and statistical power are not immediately applicable. The review comprised 16 research and provided a useful evidence base to map the literature. However, the few qualifying research, especially those examining cost-effectiveness, restrict the robustness of any conclusions that may be derived in terms of economic consequences. Thus, the sufficiency of sample size must be evaluated in terms of coverage of the evidence rather than statistical power.

**AUTHOR RESPONSE**

**We agree with the reviewer’s comment. In a scoping review, the volume of included literature reflects the landscape of existing evidence rather than statistical power, and the severe lack of economic evaluations inherently limits the strength of any economic conclusions we can draw. This is captured in the discussion section as a limitation, acknowledging that the extreme scarcity of economic studies limits the robustness of conclusions about the economic consequences and value for money of modern WASH interventions, further underscoring it as a primary knowledge gap identified by our review, see lines 449-471.**

-Yes, in general.
The authors used descriptive and narrative synthesis approaches suitably according to approved scoping review methodology. Given the variety in the study designs, interventions, and outcome measures, this review does not attempt to quantitatively pool or meta-analyze the results. In general, the findings are supported by the evidence. However, more rigorous comparative synthesis across studies and structured evaluation of the efficacy of the intervention would further improve the analytical quality of the text.

**AUTHOR RESPONSE**

**We appreciate the reviewer’s insightful comment on the synthesis’s analytical depth. While we agree that highlighting comparative patterns is important, we chose not to conduct a structured evaluation of effectiveness or a risk-of-bias synthesis across the studies.**

**Following the Arksey and O’Malley framework and PRISMA-ScR guidelines, the main goal of this scoping review is to outline and examine the existing literature and spot structural evidence gaps, such as the significant lack of economic data. Trying to conduct a thorough evaluation of intervention effectiveness across such varied study designs, target populations, and transmission contexts could exceed the scope of a scoping review and enter the territory of a systematic review.**

**However, to improve clarity as suggested, we have added a statement at the end of Section 3.3. This statement clearly places these descriptive trends within the exploratory limits of our scoping framework. We believe this keeps the necessary rigour for this type of study. See lines 323-325.**

-No serious worries.
The study is a review of published and publicly available literature, and as such, does not require ethical approval for human subjects research. Funding, competing interests and data availability declarations are declared by the authors. No ethical or regulatory issues were detected. However, the review protocol registration information may be enhanced by giving a direct and comprehensive registration link to increase openness and reproducibility.

**AUTHOR RESPONSE**

**We have updated the opening paragraph of Section 2.0 (Materials and Methods) to replace the generic placeholder link with the direct, comprehensive Open Science Framework (OSF) registration URL where our protocol is publicly available.**

Reviewer #5: The analysis presented matches the analysis plan described in the methods. The results are organized logically, covering geographical distribution, variation in interventions, comparative effectiveness of WASH components, school versus community level interventions, species-specific effects, and evidence gaps. The findings are presented clearly with appropriate use of counts and percentages. The authors effectively highlight key patterns including the stronger evidence for Ascaris lumbricoides compared with Trichuris trichiura and hookworm, as well as the limited impact of education only interventions. Figure one, the PRISMA flow diagram, and supplementary table S2 are of sufficient quality and clarity.

**AUTHOR RESPONSE**

**We sincerely thank the reviewer for their positive feedback and for validating our Results section, structure, and supplementary materials. We are glad that the organisation, presentation of descriptive metrics, and emphasis on species-specific patterns and evidence gaps meet the journal's standards.**

Reviewer #6: -Yes. The analysis is compatible with the stated objectives and with the scoping review approach. The narrative synthesis is sufficiently answering the research questions and is in line with the stated review methodology.

**AUTHOR RESPONSE**

**We thank the reviewer for their positive feedback. We are pleased to note that the analytical depth, narrative synthesis, and alignment with the primary scoping review objectives were found to be clear, appropriate, and fully responsive to our core research questions. We appreciate the reviewer’s validation of our methodology.**

-Mostly, yes. The results are well structured and related to the aims of the study. The key conclusions and gaps in evidence are well presented although the synthesis across research may be improved.

**AUTHOR RESPONSE**

**We thank the reviewer for their positive appraisal of our structure, key conclusions, and the identification of evidence gaps. Based on other reviewers’ comments and scope of the study design, we have improved the synthesis across research studies throughout the document.**

-Yes. Yes. Figures, tables, and additional information are generally clear and acceptable. However, more comparison summary tables would be helpful in the presentation and interpretation of the data.

**AUTHOR RESPONSE**

**We thank the reviewer for their positive assessment of the clarity of our figures, tables, and supplementary files. We appreciate the suggestion that comparison summary tables could aid data presentation; however, we have respectfully elected to maintain our current narrative and descriptive synthesis approach without adding further comparative tables.

General comment:**
The results are clearly presented and supported by a suitable analytical technique. Minor adjustments to comparative synthesis and data visualisation would further enrich the work.

**AUTHOR RESPONSE**

**We are very grateful to the reviewer for their positive assessment of the clarity of our results and for validating that our analytical approach is highly suitable for this study.**

**Regarding the suggestion to incorporate additional comparative synthesis and data visualisation, we have respectfully decided to maintain the current narrative and graphical presentation. The 16 included research papers represent an exceptionally heterogeneous mix of methodologies (spanning cluster-randomised trials, observational cross-sectional studies, and mathematical modelling) and vastly different intervention types (ranging from school-based hygiene education to community-wide sanitation infrastructure). Further specialised data visualisation or rigid comparative metrics could inadvertently imply standardised baselines that do not exist across these contexts.**

**In keeping with the Arksey and O’Malley framework and PRISMA-ScR guidelines, our focus remains on mapping the presence, extent, and coverage of the existing literature while clearly demarcating structural evidence gaps. We believe our current layout, alongside the detailed data extraction matrix in Supplementary Table S2, provides the most transparent and methodologically grounded representation of the data without introducing structural biases. We sincerely thank the reviewer for their encouraging validation of our core findings.**

Reviewer #5: The conclusions are supported by the data. The authors appropriately note that integrated WASH and mass drug administration can enhance control of Ascaris lumbricoides, but evidence for other species remains inconsistent and cost-effectiveness evidence is very limited. The limitations of the analysis are clearly described, including the lack of formal economic evaluations, the absence of pilot testing for study screening, and the focus on only key at-risk groups. The authors discuss how these findings can help advance the field by guiding future research toward cost-effectiveness analyses and long-term assessments. Public health relevance is addressed through the emphasis on sustainable strategies to reduce reinfection and support the WHO 2030 neglected tropical disease targets.

**AUTHOR RESPONSE**

**We sincerely thank the reviewer for their thorough evaluation and for explicitly validating our conclusions, study limitations, and public health framing. We are glad that our efforts to transparently delineate the current boundary of STH-WASH evidence, including species-specific variations and the clear need for contemporary economic evaluations, resonated. We also appreciate the reviewer's recognition of our study’s alignment with the WHO 2030 neglected tropical disease roadmap. We are grateful for these encouraging and supportive remarks.**

Reviewer #6: -Yeah. The evidence presented in the review generally supports the findings. The authors are correct in concluding that there is potential for better STH control outcomes using WASH interventions and MDA in combination, but they also note out that is limited research on cost-effectiveness.

**AUTHOR RESPONSE**

**We thank the reviewer for their positive evaluation and for validating the core conclusions of our review. We are glad the reviewer agrees that our findings are well supported by the mapped evidence, particularly the clear potential of integrated WASH and MDA strategies and the critical gap in contemporary cost-effectiveness data. We sincerely appreciate the supportive feedback.**

-Yes. The main limitations are discussed extensively, including the small number of economic evaluations, the heterogeneity of the research covered, and methodological constraints of the review process.

**AUTHOR RESPONSE**

**We thank the reviewer for their positive evaluation and for validating the depth and transparency of our Limitations section. We are glad that our thorough discussion of the scarcity of economic evaluations, the inherent heterogeneity of the mapped studies, and our methodological constraints was found to be clear, robust, and well-balanced. No changes were requested for this section, and we sincerely appreciate the supportive feedback.**

-Yes. The analysis highlights important information gaps and suggests priorities for future research, particularly in relation to the need for more rigorous economic evaluations and long-term evaluations of integrated WASH interventions.

**AUTHOR RESPONSE**

**We sincerely thank the reviewer for their positive evaluation and for validating the core contributions of our scoping review. We are delighted that our analysis highlighted critical gaps in the global literature and that our suggested priorities for future research, specifically the urgent need for rigorous economic evaluations and long-term longitudinal assessments of integrated WASH-MDA interventions, resonated strongly. We appreciate the reviewer's supportive feedback and encouragement.**

-Yes. The public health significance is clearly articulated throughout the essay. The findings are especially relevant to inform STH control strategies and policy decisions in endemic low- and middle-income settings.

**AUTHOR RESPONSE**

**We sincerely thank the reviewer for this highly encouraging assessment and for validating the public health significance of our work. We are delighted that our findings are recognised as directly relevant to informing STH control strategies and policy frameworks in endemic low- and middle-income settings, which was a primary objective of this study. We greatly appreciate the reviewer's supportive feedback and positive endorsement of our manuscript.**

Reviewer #5: I recommend minor revision. The authors should correct two remaining issues. First, they must standardize the reference list to a single consistent format according to journal guidelines. This requires adding missing journal names and using either all abbreviated or all full journal titles throughout. Second, they should expand the grey literature search description in the methods section to include specific details such as the names of organizations and conferences searched, or alternatively clarify that no grey literature met the inclusion criteria. No other changes are needed.

**AUTHOR RESPONSE**

**We thank the reviewer for their positive evaluation of our scoping review and for highlighting these important areas for improvement. We have addressed both minor issues in the updated version of the manuscript as detailed below:**

**- We have revised Section 2.3 (Databases searched) to explicitly list the specific organisational websites, institutional repositories, and conference proceedings that were manually searched.**

**- We have revised the reference list to correct all formatting inconsistencies.**

Reviewer #6: The manuscript is generally well ordered and enhanced greatly. But there are a few editorial and presentation problems to be sorted out before it is published.

**AUTHOR RESPONSE**

**We sincerely thank the reviewer for their positive assessment and note that the manuscript is well-ordered and significantly enhanced. We completely agree that maintaining a flawless editorial presentation is critical prior to final publication. We have made editorial and presentation enhancements to the manuscript, including the references section, to ensure it is ready for publication.**

-Transparency and reproducibility should be further enhanced by providing more material on the search technique, preferably as a supplementary file.

**AUTHOR RESPONSE**

**We fully agree with the reviewer that maximising transparency and absolute reproducibility is paramount to a rigorous scoping review.**

**To address this concern, we had detailed our search method in our formal, standalone Study Protocol, including the exact, unabbreviated Boolean search strings, full keyword combinations, specific database filters, and the institutional repositories screened.**

**As explicitly stated in the Methods section of the primary text, this comprehensive resource is publicly available as an open-access supplementary repository on the Open Science Framework (OSF). This ensures that all researchers have immediate, unfettered access to the exact operational architecture of our search technique, enabling flawless replication and perfectly aligning with the reviewer’s suggestion.**

-Summary tables highlighting the features of the included studies, intervention components, and significant results will increase readability and help with comparisons between studies.

**AUTHOR RESPONSE**

**We thank the reviewer for this comment. We appreciate the suggestion that summary tables could improve readability and facilitate comparisons between studies; however, we have respectfully elected to maintain our current narrative and descriptive synthesis approach without adding further comparative tables.**

-Evidence on cost-effectiveness is poorly characterized. Only one formal economic evaluation was found, although a more structured explanation of economic findings would add strength to the work.

**AUTHOR RESPONSE**

**We fully agree with the reviewer that evidence on cost-effectiveness is scarce and poorly characterised in the current literature. This acute shortage is among the most significant structural gaps identified in our scoping review.**

**In response to the suggestion to provide a more structured synthesis or expanded explanation of these economic findings, we have respectfully elected to maintain our current presentation without further modifications to the text. Because our exhaustive search strategy yielded exactly one formal economic evaluation meeting the inclusion criteria, there is an absolute lack of comparative data points to structure or synthesise. Attempting to build a broader economic framework or a dedicated analysis section around a single study would risk overinterpreting an isolated data point and assigning it disproportionate weight, which runs counter to the mapping mandate of a scoping review.**

**To ensure this issue is transparently addressed for the reader, we have already extensively discussed the poorly characterised nature of the economic data and the constraints of finding only one formal economic evaluation in the expanded Limitations paragraph of the Discussion section. We believe this framing accurately and responsibly reflects the true state of the global literature without introducing structural bias. We thank the reviewer for highlighting the critical importance of this gap.**

-Minor language editing is suggested to improve sentence flow and uniformity of terminology in the paper, especially when referring to different STH species and intervention categories

**AUTHOR RESPONSE**

**We thank the reviewer for this valuable suggestion to ensure the utmost precision in our terminology and language flow. We fully agree that strict uniformity is essential when discussing distinct parasite species and multi-component interventions.**

**We have conducted a thorough, line-by-line language and editorial audit of the manuscript to ensure complete consistency. Specifically, we verified that all soil-transmitted helminth (STH) parasites, including Ascaris lumbricoides, Trichuris trichiura, and hookworms, are uniformly referred to by their standard scientific names throughout the text. Furthermore, we have harmonised the descriptions of all intervention categories, ensuring that mass drug administration (MDA) and water, sanitation, and hygiene (WASH) components are clearly defined, categorised, and referenced with absolute uniformity across all sections. These edits have streamlined sentence flow and enhanced the paper’s structural clarity.**

- All additional resources, tables, and figures should be referred to consistently in the primary text to aid reader navigation.

**AUTHOR RESPONSE**

**We thank the reviewer for this suggestion to improve navigation of our manuscript. We have conducted a thorough review of the text and ensured that every figure, table, and supplementary resource is explicitly, consistently, and prominently cross-referenced.**

Reviewer #5: This scoping review addresses a timely and policy-relevant topic. The authors have successfully synthesized evidence from sixteen studies and identified critical gaps in the literature, most notably the near absence of cost-effectiveness analyses for WASH interventions in STH control. The manuscript is well written, and the conclusions are appropriately cautious. The strengths include a systematic approach, clear identification of species-specific intervention effects, and practical implications for policymakers. The weaknesses are limited to minor technical issues concerning reference formatting and grey literature search transparency. With these two corrections made, the manuscript will be suitable for publication. I recommend minor revision.

**AUTHOR RESPONSE**

**We thank the reviewer for their positive evaluation of our scoping review and for highlighting these important areas for improvement. We have addressed both minor issues in the updated version of the manuscript as detailed below:**

**- We have revised Section 2.3 (Databases searched) to explicitly list the specific organizational websites, institutional repositories, and conference proceedings that were manually searched.**

**- We have revised the reference list to correct all formatting inconsistencies.**

Reviewer #6: This publication addresses an important policy-relevant issue in the area of neglected tropical diseases, i.e., assessing the impact and cost-effectiveness of WASH interventions for control and elimination of soil-transmitted helminthiasis. The topic matter is timely and very relevant to current global efforts to attain the WHO 2030 NTD targets.

**AUTHOR RESPONSE**

**We sincerely thank the reviewer for their encouraging summary and for affirming the public health and policy relevance of our scoping review. We are glad that the focus on mapping both the epidemiological impact and the cost-effectiveness data for integrated WASH strategies was recognised as timely and highly aligned with ongoing global efforts to achieve the WHO 2030 NTD targets. We greatly appreciate the reviewer's supportive feedback and positive endorsement of our manuscript.**

The study is based on a well-established methodology of a scoping review according to the PRISMA-ScR reporting guidelines, with a heavy focus on identification of evidence gaps. The findings are significant for providing a review of the existing data and for showing the potential usefulness of integrating WASH activities with mass medication distribution, especially for the prevention of reinfection with Ascaris lumbricoides. The paper also underscores the essential lack of economic evaluations on this issue, an important addition for researchers and policymakers.

**AUTHOR RESPONSE**

**We are very grateful to the reviewer for this encouraging summary and for the positive validation of our work. We are delighted that the structural alignment with the PRISMA-ScR guidelines and our explicit focus on mapping evidence gaps were well received. We are also glad the reviewer emphasised the public health importance of our species-specific findings on *Ascaris lumbricoides*, as well as the value of highlighting the critical shortage of contemporary economic evaluations for both the research and policy communities. We sincerely thank the reviewer for their time, thorough review, and supportive feedback.**

The revised paper addresses many of the problems mentioned in the peer review. The manuscript is clearer and better organised, and the interpretation of the data has improved. However, significant limitations remain, including the relatively restrictive search technique, the little economic evidence available for synthesis, and the mostly descriptive nature of parts of the analysis.

**AUTHOR RESPONSE**

**We sincerely thank the reviewer for recognising the significant improvements in clarity, organisation, and data interpretation in our revised manuscript.**

**We fully agree that these structural characteristics persist in the final work. However, we respectfully note that the scarcity of economic evaluations, the deep heterogeneity of the data, and the resulting descriptive nature of the synthesis are not flaws in the review design, but rather the primary, foundational baseline findings of the scoping review itself. The primary mandate of this study type is to chart the exact boundaries of the existing literature landscape, and in doing so, we have transparently exposed these precise gaps, particularly the critical lack of contemporary cost-effectiveness evaluations.**

**To ensure these boundaries are clear to the reader, we have fully articulated the constraints of the search architecture, data availability, and descriptive synthesis within the expanded Limitations paragraph of the Discussion section. We highly appreciate the reviewer’s constructive feedback throughout this peer-review cycle, which has vastly improved the transparency and structural rigor of our paper.**

In sum, the book is a useful addition to the area and gives crucial insights for future research, policy development, and program implementation. I have no serious concerns about research ethics, publication ethics, or duplicate publication. The manuscript is worthy of publication with minor editorial changes and improved presentation.

**AUTHOR RESPONSE**

**We are incredibly grateful to the reviewer for this encouraging summary and for recommending our manuscript for publication. We are delighted that the review is considered a useful addition to the field and that its insights are recognised as crucial for shaping future research, policy development, and program implementation globally. We have meticulously addressed all minor editorial and presentation suggestions throughout the text to ensure the final manuscript meets the journal's highest standards.**
