# Supplementary material for: Impact and cost-effectiveness of water, sanitation, hygiene, and health education interventions in the control and elimination of soil-transmitted helminthiasis: A scoping review
Source: PLoS Negl Trop Dis. 2026 Aug 25;20(8):e0014664. doi: 10.1371/journal.pntd.0014664 (PMC13529219; doi:10.1371/journal.pntd.0014664)
Supplement: Supplementary file 7 [file pntd.0014664.s008.docx]

Reviewer #5: Revision 3 has quietly but decisively resolved the substantive methodological concerns that lingered from earlier rounds, and the manuscript now stands on firmer ground. The study objective is crisp and well suited to a scoping review, with no awkward reaching for a statistical hypothesis where none belongs. The authors have threaded a genuinely helpful conceptual framework through the introduction, showing how mass drug administration quiets the worm burden within the individual body while WASH and health education snap the environmental chains that perpetuate reinfection. The choice of the Arksey and O'Malley framework, aligned with PRISMA-ScR reporting principles, feels inevitable rather than merely appropriate; it is precisely the right tool for mapping a literature this tangled and heterogeneous. The focus on children and women of reproductive age mirrors the priorities of global STH programmes, and the authors earn trust by openly acknowledging that this lens cannot capture the messier dynamics of whole-community transmission. The sixteen studies are not a statistically powered sample but simply the known world of evidence meeting the eligibility criteria, and the scarcity of economic evaluations is worn not as a shortcoming but as a sobering finding in its own right. The narrative synthesis is the only intellectually honest path through thickets of mismatched designs, populations, parasites, and outcome definitions, and the authors wisely resist any temptation toward meta-analytical overreach. A handful of threads still need tying before production: the exact dates the searches were last run, a candid reconciliation between the stated intention to search grey literature and the restriction to peer-reviewed sources, and a clear declaration of whether any grey records actually crossed the finish line into the review. No ethical shadows fall across this work, as it harvests only what is already published and publicly available.

**AUTHOR RESPONSE**

***We thank the reviewer for highlighting these important points on reporting transparency. We have addressed each of them as follows:***

1. ***Search Execution Window: Because the literature searches were conducted collaboratively across the author team, individual searches were completed at slightly different times. We have clarified in Section 2.3 that all database and repository searches were completed between June and September 2025.***
2. ***Grey Literature Criteria Reconciliation: We have revised the wording in Section 2.2 to clarify that both peer-reviewed journal articles and grey literature reports were eligible, provided they met our Population-Concept-Context (PCC) criteria. We have also added a statement in Section 3.1 noting that, although grey literature repositories and conference proceedings were systematically searched, no grey literature records met all eligibility criteria for inclusion in the final review. Consequently, all 16 included studies in the final synthesis are peer-reviewed publications.***

Reviewer #5: The Results section breathes with a quiet architectural logic, moving the reader through geographical spread, study design, intervention type, delivery at school versus community level, parasite-specific outcomes, and the near silence of economic data. The descriptive counts and percentages sit cleanly on the page, never oversold, and the authors draw a steady line between the relatively consistent signal for Ascaris lumbricoides and the faint, contradictory whispers surrounding Trichuris trichiura and hookworm. A particularly sober and important insight surfaces when the data reveal that shifts in knowledge and hygiene behaviour can collapse into epidemiological irrelevance when the physical scaffolding of water and sanitation hardware is absent. The absence of statistical pooling is a feature rather than a flaw, allowing both positive and null findings to carry their full and honest weight without being crushed into a misleading composite. Economic evidence remains a ghost at the table, with a single formal evaluation haunting the entire evidence map. Figure 1 and the supplementary study-characteristics file do their quiet work well, though the flow diagram deserves a final careful gaze to ensure it traces every search pathway, including grey literature and citation chasing, and plainly names the reasons manuscripts fell at the full-text gate. The section stands clear and complete, ready to carry its findings toward publication.

**AUTHOR RESPONSE**

***We are deeply grateful to the reviewer for this thoughtful and highly encouraging evaluation of our Results section. Regarding Figure 1, we have conducted a final review in light of the reviewer’s comments. We would like to clarify that it represents the consolidated flow of all search pathways, including database searches (PubMed, Google Scholar) and grey literature repositories. To maintain visual clarity and prevent the flow diagram from becoming overly cluttered, we have retained the concise graphical structure of Figure 1. We thank the reviewer for helping us ensure complete reporting transparency across the text and supporting materials.***

Reviewer #5: The conclusions walk a tightrope between cautious optimism and honest restraint, and they never lose their balance. There is no sweeping declaration that integrated WASH and mass drug administration works universally; instead, the authors offer a measured recognition that these approaches hold genuine potential for Ascaris lumbricoides while remaining stubbornly inconsistent for other species and settings. The limitations are laid on the table without defensiveness: the hollow space where contemporary cost-effectiveness analyses ought to sit, the reliance on two bibliographic platforms, the absence of formal inter-reviewer reliability testing, and the leap required to stretch school-based findings across entire communities. Yet from these very gaps, a generative and actionable research agenda rises naturally, calling for long-term evaluations, community-level implementation science, rigorous coverage metrics, standardized economic analyses, and study designs sharp enough to tease the effect of concrete infrastructure apart from the whisper of behaviour change. The public health stakes need no exaggeration, given the stubborn entanglement of soil-transmitted helminthiasis with poverty, inadequate sanitation, post-chemotherapy reinfection, and the quiet theft of children's health and educational futures. The manuscript lands squarely in the conversation that matters most, feeding directly into the evidence appetite of the WHO 2030 neglected tropical disease targets.

**AUTHOR RESPONSE**

***We are deeply grateful to the reviewer for this positive, thoughtful, and encouraging feedback. We appreciate the reviewer’s recognition of our efforts to present a balanced synthesis that highlights both the promise of integrated WASH/MDA strategies and the clear limitations of the current evidence base, particularly in economic evaluations and parasite-specific nuances. We share the reviewer’s conviction that addressing these critical evidence gaps is vital to advancing towards the WHO 2030 NTD elimination goals.***

Reviewer #5: The manuscript requires only minor technical corrections before acceptance, with no need for new analyses or further external review. The reference list must be converted to the journal's numbered Vancouver style, ensuring all cited studies appear, including Clarke et al. 2018, Ercumen et al. 2019, Gizaw et al. 2019, and Pickering et al. 2019, while correcting the "Freeman et al. 2013b" citation, resolving "n.d." entries, standardizing WHO authorship, restoring missing bibliographic details, and applying consistent NLM abbreviations. Final search dates for each database and grey-literature sources must be reported, and the authors should explicitly state whether grey literature was eligible and whether any records were included, with Figure 1 reflecting all search pathways or clearly noting the absence of eligible grey records. The Author Summary requires shortening to 150 to 200 words in accessible first-person language without self-evaluative phrasing. Minor formatting corrections include restoring "PRISMA-ScR," standardizing T. trichiura spacing, removing the comma in "Okoyo, et al.," and ensuring terminological consistency throughout. The Data Availability Statement should read as a single sentence confirming all data are provided within the manuscript and Supporting Information, and response-letter line references must be synchronized with the final manuscript.

**AUTHOR RESPONSE**

***We thank the reviewer for this valuable feedback. We have updated the reference list to follow the journal’s Vancouver style and added all missing citations. We have also resolved all “n.d” entries (see the references section). As the database searches were conducted by different members of the team, we have included the start and end dates of the search (see section 2.3: Databases Searched). We have also shortened the author summary to 182 words. The data availability statement has been revised as requested (see lines 377-379). The grey literature sources have been updated (see section 2.3: Databases Searched). We have also indicated that none of the grey literature met the inclusion criteria for the final review (see lines 227 – 229).***

Reviewer #6: The manuscript is generally well written. Minor editorial revision is recommended including careful proofreading for consistency of language, correction of a few grammatical and typographical errors, and minor improvements in the clarity of selected tables and figure legends where appropriate. No need for big changes in data presentation.

**AUTHOR RESPONSE**

***We thank the reviewer for their positive evaluation and constructive feedback. In accordance with the reviewer’s recommendations:***

1. ***Proofreading & Typographical Corrections: The entire manuscript has undergone thorough line-by-line proofreading to correct minor typographical errors, fix species abbreviation formatting, correct citation punctuation, and ensure consistent academic language throughout.***
2. ***Figure and Table Legends: We have maintained the description of the caption and legend for Figure 1 and Supplementary Files S1 and S2.***

Reviewer #5: This scoping review arrives at a timely and policy-hungry moment, addressing the intertwined impact and cost-effectiveness of water, sanitation, hygiene, and health education interventions for the control and eventual elimination of soil-transmitted helminthiasis. Its principal intellectual strength lies in weaving epidemiological and economic questions into a single evidence map rather than treating them as separate conversations, and the findings suggest that integrated WASH and mass drug administration strategies may deliver additional benefits against Ascaris lumbricoides in certain settings while effects on Trichuris trichiura and hookworm remain frustratingly inconsistent. The near-total absence of contemporary economic evaluations emerges not as a weakness of the review but as a critically important finding for governments, donors, and programme implementers contemplating substantial investments in WASH infrastructure. Revision 3 is sharper, more transparent, and more intellectually balanced than its predecessor, with the addition of a conceptual framework, an expanded grey-literature description, a strengthened discussion of community-wide transmission, and conclusions that remain appropriately cautious. The decision to forgo meta-analysis and formal comparative-effectiveness ranking is methodologically defensible, given the heterogeneity of the included studies and the fundamentally exploratory purpose of a scoping review. The remaining weaknesses are purely editorial: reference management, reporting of search dates and grey-literature yields, compliance of the Author Summary with journal requirements, and minor inconsistencies between the response letter and the clean manuscript. These matters can be resolved during final editorial processing and do not touch the central scientific contribution. I recommend acceptance without further external review.

**AUTHOR RESPONSE**

***We are deeply appreciative of the reviewer’s thoughtful, thorough, and highly encouraging final evaluation of our manuscript. We sincerely thank the reviewer for recognising its value and its alignment with global policy and research priorities.***

Reviewer #6: The manuscript deals with an important and relevant topic and provides findings that add to the current body of knowledge. Overall, the study is well designed, the methods are appropriate and the findings support the conclusions. The manuscript is well written and well organized. Minor editorial changes are recommended to enhance clarity and readability. No major scientific, methodological or ethical concerns were identified. The overall manuscript is suitable for publication with minor revision.

**AUTHOR RESPONSE**

***We sincerely thank the reviewer for their positive evaluation, encouraging feedback, and recommendation for publication. We appreciate the reviewer's recognition of the clarity, methodological rigour, and relevance of our study. We have conducted a thorough, line-by-line proofread of the entire manuscript to refine sentence structure, correct minor typographical errors, standardise formatting, and improve overall readability for final publication.***
