# Supplementary material for: Impact and cost-effectiveness of water, sanitation, hygiene, and health education interventions in the control and elimination of soil-transmitted helminthiasis: A scoping review
Source: PLoS Negl Trop Dis. 2026 Aug 25;20(8):e0014664. doi: 10.1371/journal.pntd.0014664 (PMC13529219; doi:10.1371/journal.pntd.0014664)
Supplement: Peer review history

# Peer review: Decision Letter 0

**DOI:** 10.1371/journal.pntd.0014664.r001

**Authors:** Maria Victoria Periago, Mohammad Jokar

23 Dec 2025

Impact and Cost-Effectiveness of Water, Sanitation, Hygiene, and Health Education Interventions in the Control and Elimination of Soil-Transmitted Helminthiasis: A Scoping Review

PLOS Neglected Tropical Diseases

Dear Dr. Okoyo,

Thank you for submitting your manuscript to PLOS Neglected Tropical Diseases. After careful consideration, we feel that it has merit but does not fully meet PLOS Neglected Tropical Diseases's publication criteria as it currently stands. Therefore, we invite you to submit a revised version of the manuscript that addresses the points raised during the review process.

Please submit your revised manuscript within by Feb 21 2026 11:59PM. If you will need more time than this to complete your revisions, please reply to this message or contact the journal office at plosntds@plos.org. When you're ready to submit your revision, log on to https://www.editorialmanager.com/pntd/ and select the 'Submissions Needing Revision' folder to locate your manuscript file.

Please include the following items when submitting your revised manuscript:

* A letter that responds to each point raised by the editor and reviewer(s). You should upload this letter as a separate file labeled 'Response to Reviewers'. This file does not need to include responses to any formatting updates and technical items listed in the 'Journal Requirements' section below.

* A marked-up copy of your manuscript that highlights changes made to the original version. You should upload this as a separate file labeled 'Revised Manuscript with Track Changes'.

* An unmarked version of your revised paper without tracked changes. You should upload this as a separate file labeled 'Manuscript'.

If you would like to make changes to your financial disclosure, competing interests statement, or data availability statement, please make these updates within the submission form at the time of resubmission. Guidelines for resubmitting your figure files are available below the reviewer comments at the end of this letter.

We look forward to receiving your revised manuscript.

Kind regards,

Mohammad Jokar, DVM

Guest Editor

PLOS Neglected Tropical Diseases

María Periago

Section Editor

PLOS Neglected Tropical Diseases

Shaden Kamhawi

co-Editor-in-Chief

PLOS Neglected Tropical Diseases

orcid.org/0000-0003-4304-636XX

Paul Brindley

co-Editor-in-Chief

PLOS Neglected Tropical Diseases

orcid.org/0000-0003-1765-0002

**Additional Editor Comments:**

"Timely manner" justifies broader strategies and no double screening, but this deviates from standard scoping review rigor, potentially introducing subjectivity.

Question focuses on impact and cost-effectiveness of WASH and health education for STH control/elimination, but lacks specificity on outcomes (e.g., prevalence reduction, reinfection rates).

Target populations limited to preschool/school-aged children and women of reproductive age, excluding general populations or adults, which may miss broader WASH-STH evidence; global scope and unrestricted time appropriate for scoping; exclusion of systematic reviews/meta-analyses without primary data reasonable, but could overlook synthesized evidence; PCC alignment noted but not explicitly mapped.

Only PubMed and Google Scholar used, limiting comprehensiveness—missing specialized databases like Embase, Scopus, or Global Health; grey literature "reviewed" but no systematic search method described (e.g., specific repositories or hand-searching protocols).

Keywords comprehensive for WASH, STH species, and interventions, but OR/AND structure may yield overly broad results; inclusion of "mass drug administration" relevant for context but not core to WASH focus; no MeSH terms or synonyms for completeness (e.g., "latrine" for sanitation).

Single screening for titles/abstracts and full texts increases risk of errors/inconsistencies; Mendeley used appropriately; eligibility tied to PCC, but no mention of pilot testing or inter-rater agreement; flow diagram (Figure 1) referenced but numbers/reasons for exclusions not summarized here.

**Journal Requirements:**

If the reviewer comments include a recommendation to cite specific previously published works, please review and evaluate these publications to determine whether they are relevant and should be cited. There is no requirement to cite these works unless the editor has indicated otherwise.

1) Please upload all main figures as separate Figure files in .tif or .eps format. For more information about how to convert and format your figure files please see our guidelines:

https://journals.plos.org/plosntds/s/figures

2) We have noticed that you have uploaded Supporting Information files, but you have not included a list of legends. Please add a full list of legends for your Supporting Information files after the references list.

3) We note that your Data Availability Statement is currently as follows: "All relevant data are included within the paper.". Please confirm at this time whether or not your submission contains all raw data required to replicate the results of your study. Authors must share the “minimal data set” for their submission. PLOS defines the minimal data set to consist of the data required to replicate all study findings reported in the article, as well as related metadata and methods (https://journals.plos.org/plosone/s/data-availability#loc-minimal-data-set-definition).

For example, authors should submit the following data:

1) The values behind the means, standard deviations and other measures reported;

2) The values used to build graphs;

3) The points extracted from images for analysis..

Authors do not need to submit their entire data set if only a portion of the data was used in the reported study.

If your submission does not contain these data, please either upload them as Supporting Information files or deposit them to a stable, public repository and provide us with the relevant URLs, DOIs, or accession numbers. For a list of recommended repositories, please see https://journals.plos.org/plosone/s/recommended-repositories.

If there are ethical or legal restrictions on sharing a de-identified data set, please explain them in detail (e.g., data contain potentially sensitive information, data are owned by a third-party organization, etc.) and who has imposed them (e.g., an ethics committee). Please also provide contact information for a data access committee, ethics committee, or other institutional body to which data requests may be sent. If data are owned by a third party, please indicate how others may request data access.

**Reviewers' Comments:**

Reviewer's Responses to Questions

**Key Review Criteria Required for Acceptance?**

As you describe the new analyses required for acceptance, please consider the following:

**Methods**

-Are the objectives of the study clearly articulated with a clear testable hypothesis stated?

-Is the study design appropriate to address the stated objectives?

-Is the population clearly described and appropriate for the hypothesis being tested?

-Is the sample size sufficient to ensure adequate power to address the hypothesis being tested?

-Were correct statistical analysis used to support conclusions?

-Are there concerns about ethical or regulatory requirements being met?

Reviewer #1: Pls provide the table as the supplemental file which will reduce the length of the volume of the article

Reviewer #2: (No Response)

Reviewer #3: (No Response)

**********

**Results**

-Does the analysis presented match the analysis plan?

-Are the results clearly and completely presented?

-Are the figures (Tables, Images) of sufficient quality for clarity?

Reviewer #1: Please mention about the name of the parasites found in the included articles.

Pls mention the name of the anthelmintics used in the MDA of the included studies

Provide the results of the MDA and MDA+WASH stated in the different articles

Nothing mentioned about the cost MDAs or MDAs+WASH; Nothing is free.

Reviewer #2: (No Response)

Reviewer #3: (No Response)

**********

**Conclusions**

-Are the conclusions supported by the data presented?

-Are the limitations of analysis clearly described?

-Do the authors discuss how these data can be helpful to advance our understanding of the topic under study?

-Is public health relevance addressed?

Reviewer #1: (No Response)

Reviewer #2: (No Response)

Reviewer #3: (No Response)

**********

**Editorial and Data Presentation Modifications?**

Use this section for editorial suggestions as well as relatively minor modifications of existing data that would enhance clarity. If the only modifications needed are minor and/or editorial, you may wish to recommend “Minor Revision” or “Accept”.

Reviewer #1: (No Response)

Reviewer #2: (No Response)

Reviewer #3: Dear Editor,

Re: Reviewer Comments:

Thank you for giving me the privilege to review manuscript, “Impact and Cost-Effectiveness of Water, Sanitation, Hygiene, and Health Education Interventions in the Control and Elimination of Soil-Transmitted Helminthiasis: A Scoping Review”

Find below my comments on the MS.

1. Abstract

• The background section is inadequate and does not sufficiently contextualize the study or clearly articulate the rationale for the scoping review. The reader is not adequately oriented to the existing evidence gap.

• The sentence under Methods (“This scoping review aimed to systematically map and summarize the impact and cost-effectiveness of WASH evidence for STH control and elimination efforts.”) is more appropriate in the Background, as it states the study objective rather than a methodological procedure.

2. Discussion

• The discussion is underdeveloped and does not meet current standards for scoping reviews. The authors fail to situate their findings within existing literature, and the absence of citations limits interpretability and undermines the contribution of the review. In line with PRISMA-ScR guidance, the discussion should explicitly relate the mapped evidence to prior studies, highlight consistencies or gaps, and clarify how the findings advance the field.

3. Specific Statements for Clarification

• “However, behavioral improvements alone were insufficient without supporting infrastructure, and their effects on other helminths were inconsistent”: It is unclear whether “other helminths” refers specifically to other STH species or helminths more broadly. This distinction is important, as not all helminths are primarily driven by hygiene and sanitation conditions. The authors should clarify the scope of this statement.

• “At the household level, integrated WASH packages… led to an approximate 22% reduction in A. lumbricoides prevalence”: This quantitative statement is problematic in a scoping review context. The manuscript does not explain whether this figure comes from a single study, a pooled estimate, or descriptive synthesis. Note that, scoping reviews should avoid meta-analytic or effect-size language unless a formal quantitative synthesis is conducted. The source of this estimate should be clarified, and wording revised to reflect descriptive reporting.

**********

**Summary and General Comments**

Use this section to provide overall comments, discuss strengths/weaknesses of the study, novelty, significance, general execution and scholarship. You may also include additional comments for the author, including concerns about dual publication, research ethics, or publication ethics. If requesting major revision, please articulate the new experiments that are needed.

Reviewer #1: The topic is interesting but requires substantial modifications before acceptance

Abstract and Author summary

Pls provide the results in this sections

Introductions

WASH: stands only for water, sanitation, and hygiene; so put 'WASH' before 'and'

Pls provide a paragraph on the impacts of the STFs

Discussion

Very poorly discussed. Please discuss your findings point by point

Reviewer #2: (No Response)

Reviewer #3: The manuscript addresses an important topic, but the current version requires some revisions. Key issues include insufficient contextualization in the abstract and discussion, lack of citations to situate findings within the literature (discussion section), ambiguous statements regarding helminth effects, and inappropriate use of quantitative effect language in a scoping review. Addressing these issues will enhance the rigor, clarity, and contribution of the manuscript.

**********

PLOS authors have the option to publish the peer review history of their article (what does this mean?). If published, this will include your full peer review and any attached files.

If you choose “no”, your identity will remain anonymous but your review may still be made public.

**Do you want your identity to be public for this peer review?**  For information about this choice, including consent withdrawal, please see our Privacy Policy.

Reviewer #1: **Yes:** Anisuzzaman

Reviewer #2: No

Reviewer #3: No

[NOTE: If reviewer comments were submitted as an attachment file, they will be attached to this email and accessible via the submission site. Please log into your account, locate the manuscript record, and check for the action link "View Attachments". If this link does not appear, there are no attachment files.]

**Figure resubmission:**

While revising your submission, we strongly recommend that you use PLOS’s NAAS tool (https://ngplosjournals.pagemajik.ai/artanalysis) to test your figure files. NAAS can convert your figure files to the TIFF file type and meet basic requirements (such as print size, resolution), or provide you with a report on issues that do not meet our requirements and that NAAS cannot fix.

**Reproducibility:**

To enhance the reproducibility of your results, we recommend that authors of applicable studies deposit laboratory protocols in protocols.io, where a protocol can be assigned its own identifier (DOI) such that it can be cited independently in the future. Additionally, PLOS ONE offers an option to publish peer-reviewed clinical study protocols. Read more information on sharing protocols at https://plos.org/protocols?utm_medium=editorial-email&utm_source=authorletters&utm_campaign=protocols

Attachment Submitted filename: Review report of manuscript PNTD-D-25-01970.docx

# Author comment: Author response to Decision Letter 1

**DOI:** 10.1371/journal.pntd.0014664.r002

24 Feb 2026

Attachment Submitted filename: Response to reviewers v2.docx

# Peer review: Decision Letter 1

**DOI:** 10.1371/journal.pntd.0014664.r003

**Authors:** Maria Victoria Periago, Mohammad Jokar

31 Mar 2026

Response to Reviewers Revised Manuscript with Track Changes Manuscript

Shaden Kamhawi

co-Editor-in-Chief

PLOS Neglected Tropical Diseases

orcid.org/0000-0003-4304-636XX

Paul Brindley

co-Editor-in-Chief

PLOS Neglected Tropical Diseases

orcid.org/0000-0003-1765-0002

**********

Note: If the reviewer comments include a recommendation to cite specific previously published works, please review and evaluate these publications to determine whether they are relevant and should be cited. There is no requirement to cite these works unless the editor has indicated otherwise.

**Reviewers' comments:**

**Key Review Criteria Required for Acceptance?**

As you describe the new analyses required for acceptance, please consider the following:

**Methods**

-Are the objectives of the study clearly articulated with a clear testable hypothesis stated?

-Is the study design appropriate to address the stated objectives?

-Is the population clearly described and appropriate for the hypothesis being tested?

-Is the sample size sufficient to ensure adequate power to address the hypothesis being tested?

-Were correct statistical analysis used to support conclusions?

-Are there concerns about ethical or regulatory requirements being met?

Reviewer #3: (No Response)

Reviewer #4: The study objectives are clearly articulated and address a relevant and timely topic, namely the impact and cost-effectiveness of WASH interventions for the control of soil-transmitted helminthiasis. The use of the Arksey and O’Malley framework alongside PRISMA-ScR guidelines is appropriate for a scoping review and aligns with current methodological standards. However, there are some limitations that reduce the overall rigor of the methodology. The search strategy appears somewhat limited, as it relies primarily on PubMed and Google Scholar, which may restrict the comprehensiveness of the evidence base. In addition, the grey literature search is not described in sufficient detail to ensure reproducibility. While the authors clarified that screening was conducted independently, the absence of inter-rater reliability assessment and pilot testing reduces confidence in the consistency of study selection. The population focus is generally appropriate, although the exclusion of broader adult or community-level populations may limit the scope of the findings. The study design is suitable, but greater transparency and comprehensiveness are needed.

**********

**Results**

-Does the analysis presented match the analysis plan?

-Are the results clearly and completely presented?

-Are the figures (Tables, Images) of sufficient quality for clarity?

Reviewer #3: (No Response)

Reviewer #4: The results are generally consistent with the stated objectives and follow the described analytical approach. The findings are clearly presented and logically organized, with appropriate descriptive synthesis consistent with scoping review methodology. The manuscript does a good job of categorizing studies by design and geographic distribution, and it highlights important gaps in the literature, particularly the limited evidence on cost-effectiveness. However, the reporting of intervention details is somewhat limited, especially regarding variability in mass drug administration regimens and the specific WASH components implemented. The discussion of cost-related findings is relatively minimal and would benefit from further elaboration. In some instances, the results remain largely descriptive without sufficient comparison or synthesis across studies. The figures and tables are generally appropriate and clear, although additional comparative summaries could improve clarity and interpretation.

**********

**Conclusions**

-Are the conclusions supported by the data presented?

-Are the limitations of analysis clearly described?

-Do the authors discuss how these data can be helpful to advance our understanding of the topic under study?

-Is public health relevance addressed?

Reviewer #3: (No Response)

Reviewer #4: The conclusions are generally supported by the data presented and appropriately emphasize the potential benefits of integrating WASH interventions with mass drug administration. The manuscript effectively highlights key evidence gaps, particularly the lack of robust economic evaluations. However, the conclusions may slightly overstate the strength of the evidence given the heterogeneity of included studies and the limited number of cost-effectiveness analyses. The policy implications could be framed more cautiously in light of these limitations. Nonetheless, the public health relevance of the study is strong and clearly articulated.

**********

**Editorial and Data Presentation Modifications?**

Use this section for editorial suggestions as well as relatively minor modifications of existing data that would enhance clarity. If the only modifications needed are minor and/or editorial, you may wish to recommend “Minor Revision” or “Accept”.

Reviewer #3: (No Response)

Reviewer #4: The manuscript would benefit from improved clarity and consistency in terminology, particularly in distinguishing among different soil-transmitted helminth species. The reporting of intervention components should be strengthened to better differentiate between WASH and mass drug administration elements. The description of the grey literature search strategy should be expanded to enhance reproducibility. All supplementary materials, including tables and the PRISMA flow diagram, should be clearly referenced and labeled within the text. The manuscript is generally well written in English, although minor editing would further improve clarity.

**********

**Summary and General Comments**

Use this section to provide overall comments, discuss strengths/weaknesses of the study, novelty, significance, general execution and scholarship. You may also include additional comments for the author, including concerns about dual publication, research ethics, or publication ethics. If requesting major revision, please articulate the new experiments that are needed.

Reviewer #3: The authors have made substantial improvement to the manuscript. However, I still think the discussion is shallow and needs additional inputs.

Subject to addressing these points and the minor corrections outlined below, the manuscript is now suitable for publication.

Correct the spelling of "discrepancies" on line 219

Check that the referencing format is correct and is applied throughout the text.

Results

Note that this observation, “Freeman et al., 2013 demonstrated a 44% reduction in reinfection risk (OR: 0.56, 95% CI: 0.31–1.00)” is not considered statistically significant. - Lines 315 to 316.

Revise line 318 to:

“However, no significant effects were observed for T. trichiura or hookworm. Similarly, a community-wide trial in Timor-Leste (WASH for WORMS) achieved a 25.9 percentage-point reduction in open defecation (66.1% to 40.2%), but did not significantly reduce STH prevalence compared with MDA alone.”

Reviewer #4: This manuscript addresses an important and policy-relevant topic in the control and elimination of neglected tropical diseases. The integration of WASH interventions with deworming programs is of significant interest to researchers, practitioners, and policymakers. The study is strengthened by its use of an appropriate scoping review framework and its clear identification of key evidence gaps, particularly in relation to cost-effectiveness. The discussion has improved and provides useful context for the findings. However, the manuscript is limited by a relatively narrow search strategy, insufficient detail in the grey literature search, and a lack of depth in the synthesis of economic evidence. Some sections remain overly descriptive rather than analytical. The manuscript has merit but requires moderate to substantial revisions to improve methodological transparency, depth of analysis, and clarity before it can be considered suitable for publication.

**********

PLOS authors have the option to publish the peer review history of their article (what does this mean?). If published, this will include your full peer review and any attached files.

If you choose “no”, your identity will remain anonymous but your review may still be made public.

**Do you want your identity to be public for this peer review?**  For information about this choice, including consent withdrawal, please see our Privacy Policy.

Reviewer #3: No

Reviewer #4: No

**Figure resubmission:**

**Reproducibility:**

To enhance the reproducibility of your results, we recommend that authors of applicable studies deposit laboratory protocols in protocols.io, where a protocol can be assigned its own identifier (DOI) such that it can be cited independently in the future. Additionally, PLOS ONE offers an option to publish peer-reviewed clinical study protocols. Read more information on sharing protocols at https://plos.org/protocols?utm_medium=editorial-email&utm_source=authorletters&utm_campaign=protocols

# Author comment: Author response to Decision Letter 2

**DOI:** 10.1371/journal.pntd.0014664.r004

27 Apr 2026

Attachment Submitted filename: Response to Reviewers.docx

# Peer review: Decision Letter 2

**DOI:** 10.1371/journal.pntd.0014664.r005

**Authors:** Maria Victoria Periago, Mohammad Jokar

18 Jun 2026

Response to Reviewers Revised Manuscript with Track Changes Manuscript

Shaden Kamhawi

co-Editor-in-Chief

PLOS Neglected Tropical Diseases

orcid.org/0000-0003-4304-636XX

Paul Brindley

co-Editor-in-Chief

PLOS Neglected Tropical Diseases

orcid.org/0000-0003-1765-0002

**Reviewers' comments:**

**Key Review Criteria Required for Acceptance?**

As you describe the new analyses required for acceptance, please consider the following:

**Methods**

-Are the objectives of the study clearly articulated with a clear testable hypothesis stated?

-Is the study design appropriate to address the stated objectives?

-Is the population clearly described and appropriate for the hypothesis being tested?

-Is the sample size sufficient to ensure adequate power to address the hypothesis being tested?

-Were correct statistical analysis used to support conclusions?

-Are there concerns about ethical or regulatory requirements being met?

Reviewer #5: revision 2 represents a clear improvement over previous versions. The authors articulate the objectives of this scoping review with appropriate clarity. Their aim is to map and summarize the evidence on the impact and cost effectiveness of water, sanitation, hygiene, and health education interventions for soil transmitted helminthiasis. A formal hypothesis is not expected for a scoping review, and the study design follows the well established Arksey and O'Malley framework alongside PRISMA ScR guidelines, which is entirely appropriate. The target populations including preschool children, school aged children, and women of reproductive age are clearly described and relevant given that these groups experience the highest infection burden. The sample size of sixteen studies is sufficient to identify key evidence gaps and patterns across the literature. The descriptive synthesis is correct for this study type, and no inappropriate inferential statistics are applied. Ethical requirements are satisfied because the review synthesizes previously published data only. Two minor issues require correction. First, the grey literature search description lacks sufficient detail for reproducibility; the authors mention manual searches of organizational websites and conference proceedings but do not specify which organizations, which conferences, or the dates of those searches. Second, the reference list contains formatting inconsistencies including missing journal names in several entries and mixed use of abbreviated versus full journal titles. Both issues can be addressed with minor revisions.

Reviewer #6: -Sorta.

The aims of the study are clearly stated, and seek to map and summarize the evidence on the impact and cost-effectiveness of WASH interventions for the control and elimination of soil-transmitted helminthiasis (STH). The research issue is well stated and appropriate to the approach of a scoping review. But there is no explicit tested hypothesis. This is understandable as scoping reviews are mainly exploratory and their goal is to map the existing evidence rather than to test a hypothesis formulated a priori. However the authors may improve conceptual clarity by explicitly addressing the expected association between integrated WASH interventions and STH outcomes.

-Yes.

The stated purpose of this review is to identify, map and synthesize available data on the impact and cost-effectiveness of WASH interventions, and therefore the adoption of a scoping review approach following the Arksey and O’Malley framework and PRISMA-ScR standards is suitable. The study design is appropriate for identification of knowledge gaps and provides a broad perspective of the literature. However, the scope of the review is somewhat constrained by the narrow database selection and the not detailed enough grey literature search approach.

-Yes.

The target populations are well defined, include preschool-aged children, school-aged children and women of reproductive age and reflect the main categories addressed by WHO approved STH management programmes. These populations are extremely relevant for the purposes of the review. Nonetheless, limiting the analysis to smaller community populations may hinder the generalizability of findings, especially for the assessment of community-wide WASH interventions and transmission dynamics.

-Unable to evaluate properly / Not applicable.

As this is a scoping study, traditional considerations of sample size and statistical power are not immediately applicable. The review comprised 16 research and provided a useful evidence base to map the literature. However, the few qualifying research, especially those examining cost-effectiveness, restrict the robustness of any conclusions that may be derived in terms of economic consequences. Thus, the sufficiency of sample size must be evaluated in terms of coverage of the evidence rather than statistical power.

-Yes, in general.

The authors used descriptive and narrative synthesis approaches suitably according to approved scoping review methodology. Given the variety in the study designs, interventions and outcome measures, this review does not attempt to quantitatively pool or meta-analyze the results. In general, the findings are supported by the evidence. However, more rigorous comparative synthesis across studies and structured evaluation of the efficacy of the intervention would further improve the analytical quality of the text.

-No serious worries.

The study is a review of published and publicly available literature, and as such, does not require ethical approval for human subjects research. Funding, competing interests and data availability declarations are declared by the authors. No ethical or regulatory issues were detected. However, the review protocol registration information may be enhanced by giving a direct and comprehensive registration link to increase openness and reproducibility.

**********

**Results**

-Does the analysis presented match the analysis plan?

-Are the results clearly and completely presented?

-Are the figures (Tables, Images) of sufficient quality for clarity?

Reviewer #5: The analysis presented matches the analysis plan described in the methods. The results are organized logically covering geographical distribution, variation in interventions, comparative effectiveness of WASH components, school versus community level interventions, species specific effects, and evidence gaps. The findings are presented clearly with appropriate use of counts and percentages. The authors effectively highlight key patterns including the stronger evidence for Ascaris lumbricoides compared with Trichuris trichiura and hookworm, as well as the limited impact of education only interventions. Figure one, the PRISMA flow diagram, and supplementary table S2 are of sufficient quality and clarity.

Reviewer #6: -Yes. The analysis is compatible with the stated objectives and with the scoping review approach. The narrative synthesis is sufficiently answering to the research questions and is in line with the stated review methodology.

-Mostly, yes. The results are well structured and related to the aims of the study. The key conclusions and gaps in evidence are well presented although the synthesis across research may be improved.

-Yes. Yes. Figures, tables and additional information are generally clear and acceptable. However, more comparison summary tables would be helpful in the presenting and interpretation of the data.

General comment:

The results are clearly presented and supported by a suitable analytical technique. Minor adjustments to comparative synthesis and data visualization would enrich the work further.

**********

**Conclusions**

-Are the conclusions supported by the data presented?

-Are the limitations of analysis clearly described?

-Do the authors discuss how these data can be helpful to advance our understanding of the topic under study?

-Is public health relevance addressed?

Reviewer #5: The conclusions are supported by the data. The authors appropriately note that integrated WASH and mass drug administration can enhance control of Ascaris lumbricoides, but evidence for other species remains inconsistent and cost effectiveness evidence is very limited. The limitations of the analysis are clearly described, including the lack of formal economic evaluations, the absence of pilot testing for study screening, and the focus on only key at risk groups. The authors discuss how these findings can help advance the field by guiding future research toward cost effectiveness analyses and long term assessments. Public health relevance is addressed through the emphasis on sustainable strategies to reduce reinfection and support the WHO 2030 neglected tropical disease targets.

Reviewer #6: -Yeah. The evidence presented in the review generally supports the findings. The authors are correct in concluding that there is potential for better STH control outcomes using WASH interventions and MDA in combination, but they also note out that is limited research on cost-effectiveness.

-Yes. The main limitations are discussed extensively including the small number of economic evaluations, the heterogeneity of the research covered and methodological constraints of the review process.

-Yes. The analysis highlights important information gaps and suggests priorities for future research, particularly in relation to the need for more rigorous economic evaluations and long-term evaluations of integrated WASH interventions.

-Yes. . The public health significance is clearly articulated throughout the essay. The findings are especially relevant to inform STH control strategies and policy decisions in endemic low- and middle-income settings.

Conclusions are generally fair and relevant and the manuscript provides significant insights for researchers and public health practitioners alike.

**********

**Editorial and Data Presentation Modifications?**

Use this section for editorial suggestions as well as relatively minor modifications of existing data that would enhance clarity. If the only modifications needed are minor and/or editorial, you may wish to recommend “Minor Revision” or “Accept”.

Reviewer #5: I recommend minor revision. The authors should correct two remaining issues. First, they must standardize the reference list to a single consistent format according to journal guidelines. This requires adding missing journal names and using either all abbreviated or all full journal titles throughout. Second, they should expand the grey literature search description in the methods section to include specific details such as the names of organizations and conferences searched, or alternatively clarify that no grey literature met the inclusion criteria. No other changes are needed.

Reviewer #6: The manuscript is generally well ordered and enhanced greatly. But there are a few editorial and presentation problems to be sorted out before it is published.

-Transparency and reproducibility should be further enhanced by providing more material on the search technique, preferably as a supplementary file.

-Summary tables highlighting the features of the included studies, intervention components and significant results will increase readability and help with comparisons between studies.

-Evidence on cost-effectiveness is poorly characterized. Only one formal economic evaluation was found, although a more structured explanation of economic findings would add strength to the work.

-Minor language editing is suggested to improve sentence flow and uniformity of terminology in the paper, especially when referring to different STH species and intervention categories

-All additional resources, tables and figures should be referred consistently in the primary text for ease of navigation by readers.

These are minor points that do not affect the overall value of the manuscript.

**********

**Summary and General Comments**

Use this section to provide overall comments, discuss strengths/weaknesses of the study, novelty, significance, general execution and scholarship. You may also include additional comments for the author, including concerns about dual publication, research ethics, or publication ethics. If requesting major revision, please articulate the new experiments that are needed.

Reviewer #5: This scoping review addresses a timely and policy relevant topic. The authors have successfully synthesized evidence from sixteen studies and identified critical gaps in the literature, most notably the near absence of cost effectiveness analyses for WASH interventions in STH control. The manuscript is well written and the conclusions are appropriately cautious. The strengths include a systematic approach, clear identification of species specific intervention effects, and practical implications for policy makers. The weaknesses are limited to minor technical issues concerning reference formatting and grey literature search transparency. With these two corrections made, the manuscript will be suitable for publication. I recommend minor revision.

Reviewer #6: This publication addresses an important policy-relevant issue in the area of neglected tropical diseases, i.e. assessing the impact and cost-effectiveness of WASH interventions for control and elimination of soil-transmitted helminthiasis. The topic matter is timely and very relevant to current global efforts to attain the WHO 2030 NTD targets.

The study is based on a well-established methodology of scoping review according to the PRISMA-ScR reporting guidelines, with a heavy focus on identification of evidence gaps. The findings are significant for providing a review of the existing data and for showing the potential usefulness of integrating WASH activities with mass medication distribution, especially for the prevention of reinfection with Ascaris lumbricoides. The paper also underscores the essential lack of economic evaluations on this issue, an important addition for researchers and policymakers.

The revised paper addresses many of the problems mentioned in the peer review. The manuscript is clearer and better organised, and the interpretation of the data has improved. However, significant limitations remain, including the relatively restrictive search technique, the little economic evidence available for synthesis and the mostly descriptive nature of parts of the analysis.

In sum, the book is a useful addition to the area and gives crucial insights for future research, policy development and program implementation. I have no serious concerns about research ethics, publication ethics, or duplication publication.The manuscript is worthy of publication with minor editorial changes and improved presentation.

**********

PLOS authors have the option to publish the peer review history of their article (what does this mean?). If published, this will include your full peer review and any attached files.

If you choose “no”, your identity will remain anonymous but your review may still be made public.

**Do you want your identity to be public for this peer review?**  For information about this choice, including consent withdrawal, please see our Privacy Policy.

Reviewer #5: **Yes:** Dr.Arman Abdous DVM,MPH

Reviewer #6: No

**Figure resubmission:**

**Reproducibility:**

To enhance the reproducibility of your results, we recommend that authors of applicable studies deposit laboratory protocols in protocols.io, where a protocol can be assigned its own identifier (DOI) such that it can be cited independently in the future. Additionally, PLOS ONE offers an option to publish peer-reviewed clinical study protocols. Read more information on sharing protocols at https://plos.org/protocols?utm_medium=editorial-email&utm_source=authorletters&utm_campaign=protocols

# Author comment: Author response to Decision Letter 3

**DOI:** 10.1371/journal.pntd.0014664.r006

11 Jul 2026

Attachment Submitted filename: Response_to_reviewers_auresp_3.docx

# Peer review: Decision Letter 3

**DOI:** 10.1371/journal.pntd.0014664.r007

**Authors:** Maria Victoria Periago, Mohammad Jokar

21 Jul 2026

Response to Reviewers Revised Manuscript with Track Changes Manuscript

Shaden Kamhawi

co-Editor-in-Chief

PLOS Neglected Tropical Diseases

orcid.org/0000-0003-4304-636XX

Paul Brindley

co-Editor-in-Chief

PLOS Neglected Tropical Diseases

orcid.org/0000-0003-1765-0002

**Additional Editor Comments (if provided):**

**Reviewers' comments:**

**Key Review Criteria Required for Acceptance?**

As you describe the new analyses required for acceptance, please consider the following:

**Methods**

-Are the objectives of the study clearly articulated with a clear testable hypothesis stated?

-Is the study design appropriate to address the stated objectives?

-Is the population clearly described and appropriate for the hypothesis being tested?

-Is the sample size sufficient to ensure adequate power to address the hypothesis being tested?

-Were correct statistical analysis used to support conclusions?

-Are there concerns about ethical or regulatory requirements being met?

Reviewer #5: Revision 3 has quietly but decisively resolved the substantive methodological concerns that lingered from earlier rounds, and the manuscript now stands on firmer ground. The study objective is crisp and well suited to a scoping review, with no awkward reaching for a statistical hypothesis where none belongs. The authors have threaded a genuinely helpful conceptual framework through the introduction, showing how mass drug administration quiets the worm burden within the individual body while WASH and health education snap the environmental chains that perpetuate reinfection. The choice of the Arksey and O'Malley framework, aligned with PRISMA-ScR reporting principles, feels inevitable rather than merely appropriate; it is precisely the right tool for mapping a literature this tangled and heterogeneous. The focus on children and women of reproductive age mirrors the priorities of global STH programmes, and the authors earn trust by openly acknowledging that this lens cannot capture the messier dynamics of whole-community transmission. The sixteen studies are not a statistically powered sample but simply the known world of evidence meeting the eligibility criteria, and the scarcity of economic evaluations is worn not as a shortcoming but as a sobering finding in its own right. The narrative synthesis is the only intellectually honest path through thickets of mismatched designs, populations, parasites, and outcome definitions, and the authors wisely resist any temptation toward meta-analytical overreach. A handful of threads still need tying before production: the exact dates the searches were last run, a candid reconciliation between the stated intention to search grey literature and the restriction to peer-reviewed sources, and a clear declaration of whether any grey records actually crossed the finish line into the review. No ethical shadows fall across this work, as it harvests only what is already published and publicly available.

Reviewer #6: Objectives: Clearly stated hypothesis to be tested.

Study design: Appropriate for the objectives of the study.

Study population: Well described and appropriate.

Sample size Adequate to support the analyses.

Statistical analyses Appropriate and correctly applied.

Ethical/Regulatory requirements : No apparent concerns

**********

**Results**

-Does the analysis presented match the analysis plan?

-Are the results clearly and completely presented?

-Are the figures (Tables, Images) of sufficient quality for clarity?

Reviewer #5: The Results section breathes with a quiet architectural logic, moving the reader through geographical spread, study design, intervention type, delivery at school versus community level, parasite-specific outcomes, and the near silence of economic data. The descriptive counts and percentages sit cleanly on the page, never oversold, and the authors draw a steady line between the relatively consistent signal for Ascaris lumbricoides and the faint, contradictory whispers surrounding Trichuris trichiura and hookworm. A particularly sober and important insight surfaces when the data reveal that shifts in knowledge and hygiene behaviour can collapse into epidemiological irrelevance when the physical scaffolding of water and sanitation hardware is absent. The absence of statistical pooling is a feature rather than a flaw, allowing both positive and null findings to carry their full and honest weight without being crushed into a misleading composite. Economic evidence remains a ghost at the table, with a single formal evaluation haunting the entire evidence map. Figure 1 and the supplementary study-characteristics file do their quiet work well, though the flow diagram deserves a final careful gaze to ensure it traces every search pathway, including grey literature and citation chasing, and plainly names the reasons manuscripts fell at the full-text gate. The section stands clear and complete, ready to carry its findings toward publication.

Reviewer #6: Analysis: Consistent with the study design and analysis plan. Results: Clearly and comprehensively presented. Figures/Tables: Of sufficient quality and clarity.

**********

**Conclusions**

-Are the conclusions supported by the data presented?

-Are the limitations of analysis clearly described?

-Do the authors discuss how these data can be helpful to advance our understanding of the topic under study?

-Is public health relevance addressed?

Reviewer #5: The conclusions walk a tightrope between cautious optimism and honest restraint, and they never lose their balance. There is no sweeping declaration that integrated WASH and mass drug administration works universally; instead, the authors offer a measured recognition that these approaches hold genuine potential for Ascaris lumbricoides while remaining stubbornly inconsistent for other species and settings. The limitations are laid on the table without defensiveness: the hollow space where contemporary cost-effectiveness analyses ought to sit, the reliance on two bibliographic platforms, the absence of formal inter-reviewer reliability testing, and the leap required to stretch school-based findings across entire communities. Yet from these very gaps, a generative and actionable research agenda rises naturally, calling for long-term evaluations, community-level implementation science, rigorous coverage metrics, standardized economic analyses, and study designs sharp enough to tease the effect of concrete infrastructure apart from the whisper of behaviour change. The public health stakes need no exaggeration, given the stubborn entanglement of soil-transmitted helminthiasis with poverty, inadequate sanitation, post-chemotherapy reinfection, and the quiet theft of children's health and educational futures. The manuscript lands squarely in the conversation that matters most, feeding directly into the evidence appetite of the WHO 2030 neglected tropical disease targets.

Reviewer #6: Conclusions: The data presented support the conclusions.

Limitations Properly acknowledged.

Contribution: The findings offer significant insights and contribute to the comprehension of the subject.

Public health relevance: Sufficiently addressed.

**********

**Editorial and Data Presentation Modifications?**

Use this section for editorial suggestions as well as relatively minor modifications of existing data that would enhance clarity. If the only modifications needed are minor and/or editorial, you may wish to recommend “Minor Revision” or “Accept”.

Reviewer #5: The manuscript requires only minor technical corrections before acceptance, with no need for new analyses or further external review. The reference list must be converted to the journal's numbered Vancouver style, ensuring all cited studies appear, including Clarke et al. 2018, Ercumen et al. 2019, Gizaw et al. 2019, and Pickering et al. 2019, while correcting the "Freeman et al. 2013b" citation, resolving "n.d." entries, standardizing WHO authorship, restoring missing bibliographic details, and applying consistent NLM abbreviations. Final search dates for each database and grey-literature sources must be reported, and the authors should explicitly state whether grey literature was eligible and whether any records were included, with Figure 1 reflecting all search pathways or clearly noting the absence of eligible grey records. The Author Summary requires shortening to 150 to 200 words in accessible first-person language without self-evaluative phrasing. Minor formatting corrections include restoring "PRISMA-ScR," standardizing T. trichiura spacing, removing the comma in "Okoyo, et al.," and ensuring terminological consistency throughout. The Data Availability Statement should read as a single sentence confirming all data are provided within the manuscript and Supporting Information, and response-letter line references must be synchronized with the final manuscript.

Reviewer #6: The manuscript is generally well written. Minor editorial revision is recommended including careful proofreading for consistency of language, correction of a few grammatical and typographical errors, and minor improvements in the clarity of selected tables and figure legends where appropriate. No need for big changes in data presentation.

**********

**Summary and General Comments**

Use this section to provide overall comments, discuss strengths/weaknesses of the study, novelty, significance, general execution and scholarship. You may also include additional comments for the author, including concerns about dual publication, research ethics, or publication ethics. If requesting major revision, please articulate the new experiments that are needed.

Reviewer #5: This scoping review arrives at a timely and policy-hungry moment, addressing the intertwined impact and cost-effectiveness of water, sanitation, hygiene, and health education interventions for the control and eventual elimination of soil-transmitted helminthiasis. Its principal intellectual strength lies in weaving epidemiological and economic questions into a single evidence map rather than treating them as separate conversations, and the findings suggest that integrated WASH and mass drug administration strategies may deliver additional benefits against Ascaris lumbricoides in certain settings while effects on Trichuris trichiura and hookworm remain frustratingly inconsistent. The near-total absence of contemporary economic evaluations emerges not as a weakness of the review but as a critically important finding for governments, donors, and programme implementers contemplating substantial investments in WASH infrastructure. Revision 3 is sharper, more transparent, and more intellectually balanced than its predecessor, with the addition of a conceptual framework, an expanded grey-literature description, a strengthened discussion of community-wide transmission, and conclusions that remain appropriately cautious. The decision to forgo meta-analysis and formal comparative-effectiveness ranking is methodologically defensible, given the heterogeneity of the included studies and the fundamentally exploratory purpose of a scoping review. The remaining weaknesses are purely editorial: reference management, reporting of search dates and grey-literature yields, compliance of the Author Summary with journal requirements, and minor inconsistencies between the response letter and the clean manuscript. These matters can be resolved during final editorial processing and do not touch the central scientific contribution. I recommend acceptance without further external review.

Reviewer #6: The manuscript deals with an important and relevant topic and provides findings that add to the current body of knowledge. Overall the study is well designed, the methods are appropriate and the findings support the conclusions. The manuscript is well written and well organized. Minor editorial changes are recommended to enhance clarity and readability. No major scientific, methodological or ethical concerns were identified. The overall manuscript is suitable for publication with minor revision.

**********

PLOS authors have the option to publish the peer review history of their article (what does this mean?). If published, this will include your full peer review and any attached files.

If you choose “no”, your identity will remain anonymous but your review may still be made public.

**Do you want your identity to be public for this peer review?**  For information about this choice, including consent withdrawal, please see our Privacy Policy.

Reviewer #5: **Yes:** Dr.Arman Abdous

Reviewer #6: No

**Figure resubmission:**

**Reproducibility:**

To enhance the reproducibility of your results, we recommend that authors of applicable studies deposit laboratory protocols in protocols.io, where a protocol can be assigned its own identifier (DOI) such that it can be cited independently in the future. Additionally, PLOS ONE offers an option to publish peer-reviewed clinical study protocols. Read more information on sharing protocols at https://plos.org/protocols?utm_medium=editorial-email&utm_source=authorletters&utm_campaign=protocols

# Author comment: Author response to Decision Letter 4

**DOI:** 10.1371/journal.pntd.0014664.r008

8 Aug 2026

Attachment Submitted filename: Response_to_reviewers_auresp_4.docx

# Editor report: Decision Letter 4

**DOI:** 10.1371/journal.pntd.0014664.r009

**Authors:** Maria Victoria Periago

10 Aug 2026

Dear Dr Okoyo,

We are pleased to inform you that your manuscript 'Impact and Cost-Effectiveness of Water, Sanitation, Hygiene, and Health Education Interventions in the Control and Elimination of Soil-Transmitted Helminthiasis: A Scoping Review' has been provisionally accepted for publication in PLOS Neglected Tropical Diseases.

Before your manuscript can be formally accepted you will need to complete some formatting changes, which you will receive in a follow up email. A member of our team will be in touch with a set of requests.

Please note that your manuscript will not be scheduled for publication until you have made the required changes, so a swift response is appreciated.

IMPORTANT: The editorial review process is now complete. PLOS will only permit corrections to spelling, formatting or significant scientific errors from this point onwards. Requests for major changes, or any which affect the scientific understanding of your work, will cause delays to the publication date of your manuscript.

Should you, your institution's press office or the journal office choose to press release your paper, you will automatically be opted out of early publication. We ask that you notify us now if you or your institution is planning to press release the article. All press must be co-ordinated with PLOS.

Thank you again for supporting Open Access publishing; we are looking forward to publishing your work in PLOS Neglected Tropical Diseases.

Best regards,

Maria Victoria Periago

Section Editor

PLOS Neglected Tropical Diseases

Shaden Kamhawi

co-Editor-in-Chief

PLOS Neglected Tropical Diseases

orcid.org/0000-0003-4304-636XX

Paul Brindley

co-Editor-in-Chief

PLOS Neglected Tropical Diseases

orcid.org/0000-0003-1765-0002

***********************************************************

The authors have made all the minor revisions suggested by the last round of reviews and the manuscript is now ready for acceptance.

# Editor report: Acceptance letter

**DOI:** 10.1371/journal.pntd.0014664.r010

**Authors:** Maria Victoria Periago

Dear Dr Okoyo,

We are delighted to inform you that your manuscript, "Impact and Cost-Effectiveness of Water, Sanitation, Hygiene, and Health Education Interventions in the Control and Elimination of Soil-Transmitted Helminthiasis: A Scoping Review," has been formally accepted for publication in PLOS Neglected Tropical Diseases.

We have now passed your article onto the PLOS Production Department who will complete the rest of the publication process. All authors will receive a confirmation email upon publication.

The corresponding author will soon be receiving a typeset proof for review, to ensure errors have not been introduced during production. Please review the PDF proof of your manuscript carefully, as this is the last chance to correct any scientific or type-setting errors. Please note that major changes, or those which affect the scientific understanding of the work, will likely cause delays to the publication date of your manuscript. Note: Proofs for Front Matter articles (Editorial, Viewpoint, Symposium, Review, etc...) are generated on a different schedule and may not be made available as quickly.

Soon after your final files are uploaded, the early version of your manuscript will be published online unless you opted out of this process. The date of the early version will be your article's publication date. The final article will be published to the same URL, and all versions of the paper will be accessible to readers.

For Research Articles, you will receive an invoice from PLOS for your publication fee after your manuscript has reached the completed accept phase. If you receive an email requesting payment before acceptance or for any other service, this may be a phishing scheme. Learn how to identify phishing emails and protect your accounts at https://explore.plos.org/phishing.

Thank you again for supporting open-access publishing; we are looking forward to publishing your work in PLOS Neglected Tropical Diseases.

Best regards,

Shaden Kamhawi

co-Editor-in-Chief

PLOS Neglected Tropical Diseases

Paul Brindley

co-Editor-in-Chief

PLOS Neglected Tropical Diseases
